# Supplementary material for: Schizophrenia-associated somatic copy-number variants from 12,834 cases reveal recurrent NRXN1 and ABCB11 disruptions
Source: Cell Genom. 2023 Jul 6;3(8):100356. doi: 10.1016/j.xgen.2023.100356 (PMC10435376; doi:10.1016/j.xgen.2023.100356)
Supplement: Document S1. Figures S1–S5 and Table S1 [file mmc1.pdf]

Cell Genomics, Volume *3*

Supplemental information

Schizophrenia-associated somatic copy-number variants from 12,834 cases reveal recurrent *NRXN1* and *ABCB11* disruptions

Eduardo A. Maury, Maxwell A. Sherman, Giulio Genovese, Thomas G. Gilgenast, Tushar Kamath, S.J. Burris, Prashanth Rajarajan, Erin Flaherty, Schahram Akbarian, Andrew Chess, Steven A. McCarroll, Po-Ru Loh, Jennifer E. Phillips-Cremins, Kristen J. Brennand, Evan Z. Macosko, James T.R. Walters, Michael O'Donovan, Patrick Sullivan, Psychiatric Genomic Consortium Schizophrenia and CNV workgroup, Brain Somatic Mosaicism Network, Jonathan Sebat, Eunjung A. Lee, and Christopher A. Walsh

Supplemental Figures:

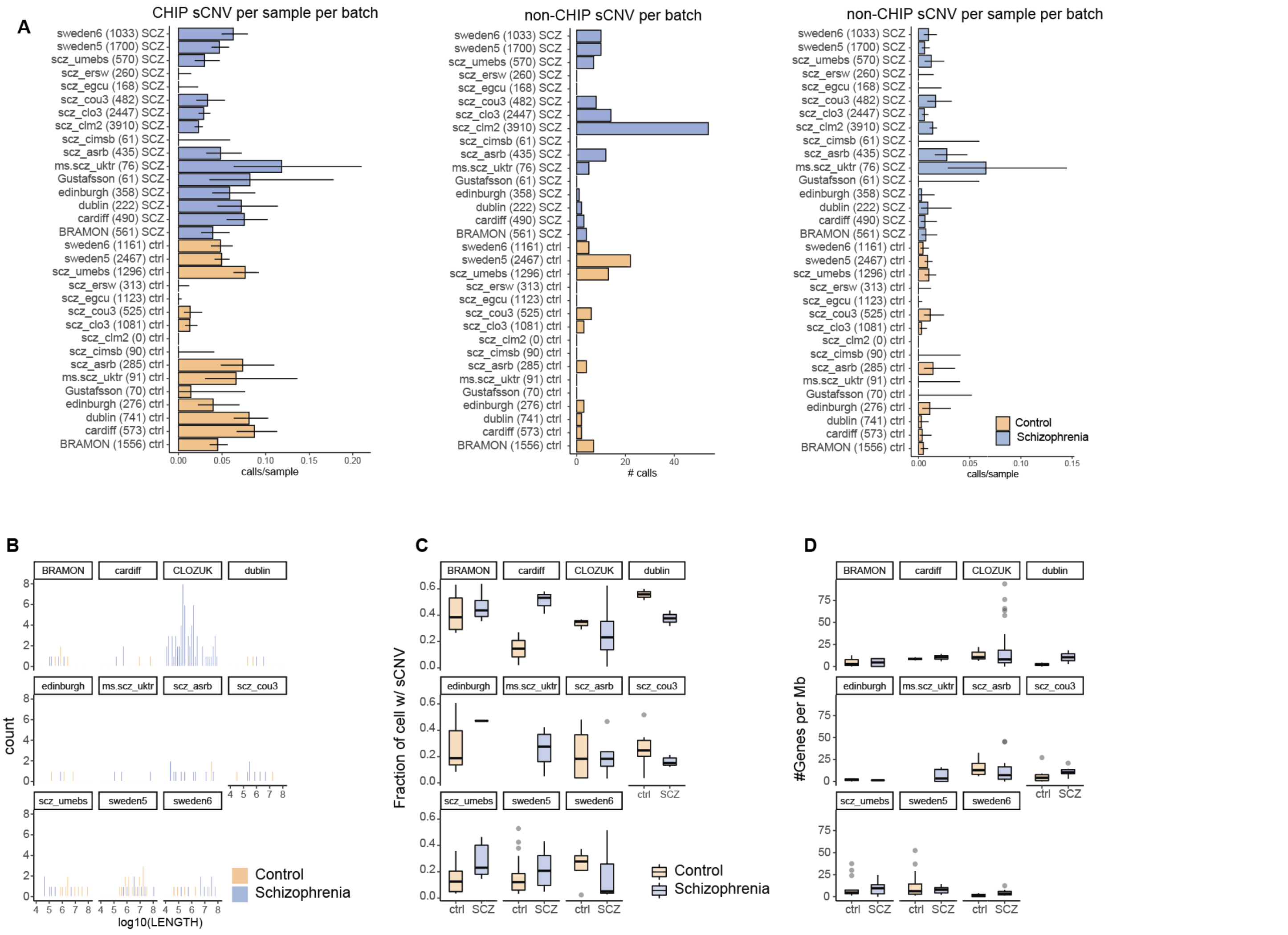

Figure S1: Characteristics of sCNVs callset across batches, related to Figure 1. A) Bar plots and forest plots of the number of sCNVs and fraction of samples with more than one sCNV in cases and controls for all batches of the data for CHIP and non-CHIP events. The number of samples on each batch is indicated in the parenthesis of the y-axis labels. The 95% confidence intervals were calculated using the Wilson's score interval with Newcombe modification. B) Histograms of sCNV length across batches for cases and controls. C) Box-plots of the fraction of cells with events (CF) in SCZ vs controls across all batches with events. D) Box-plots of the number of genes affected per megabase (Mb) in SCZ vs controls across all batches with events.

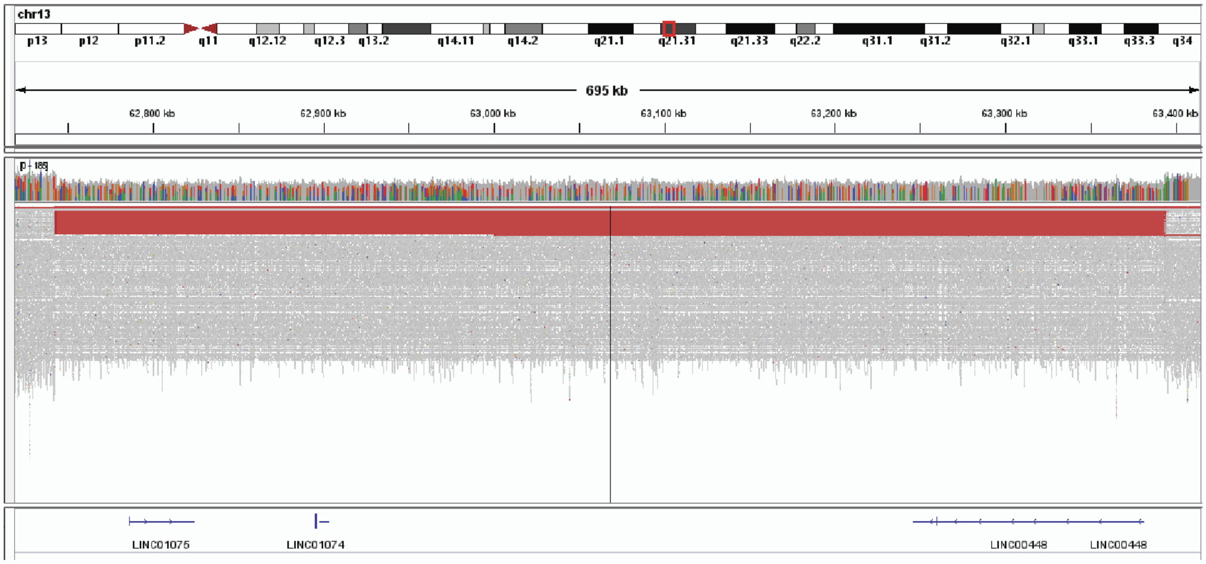

Figure S2: Further validation of sCNV, related to Figure 1. IGV plots of 9q21.11 locus. Red bars are reads with discrepant insert size corresponding to somatic deletions. The tracks from top to bottom on each panel indicates the coverage, reads mapping to that region, and RefSeq gene names respectively.

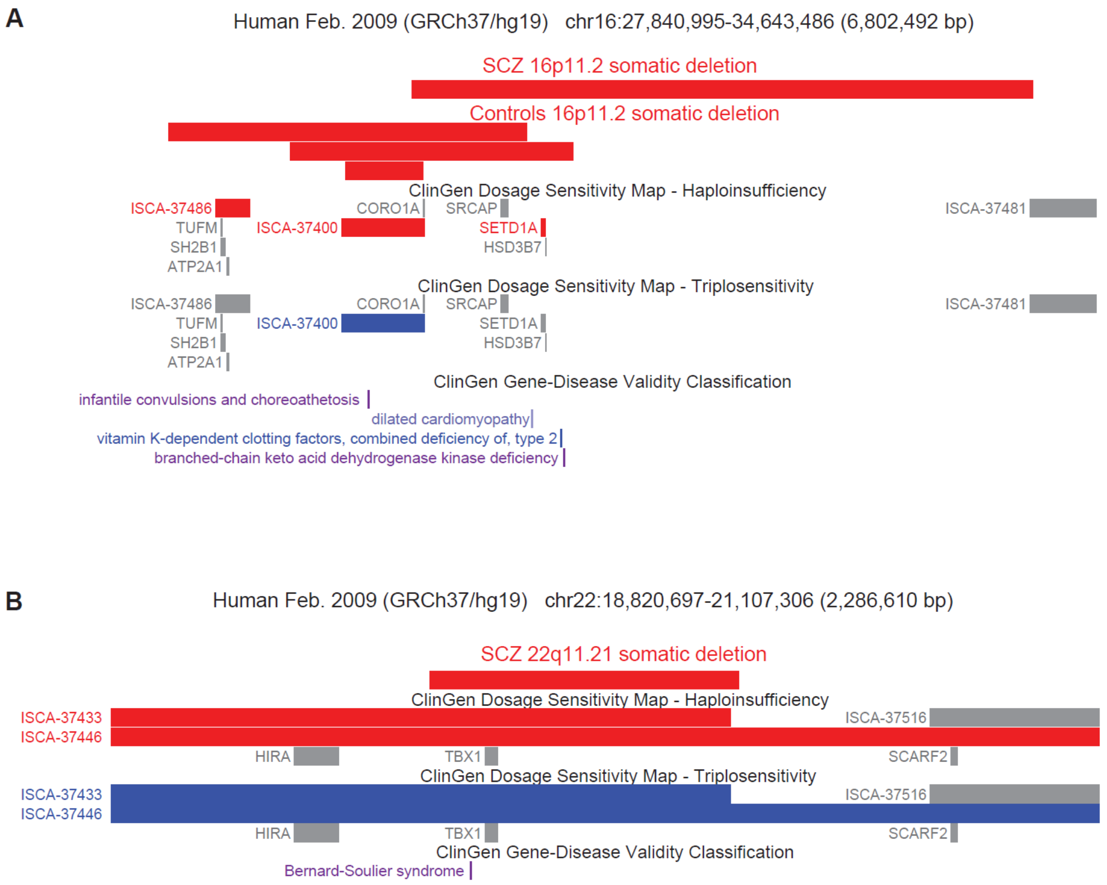

Figure S3: Somatic CNVs in 16p11.2 and 22q11.21, related to Figure 1. A) Adapted GenomeBrowser plot of 16p11.2 somatic deletions in cases and controls. Clinically relevant haploinsufficient and triplosensitive regions were annotated using the ClinGen database. Canonical 16p11.2 deletion regions are annotated by ClinGen haploinsufficiency at the proximal (ISCA-37400) and distal (ISCA-3786) sites. B) Adapted GenomeBrowser plot of 22q11.21 deletions in SCZ cases. The canonical 22q11.2 deletion regions are annotated as ISCA-37433 and ISCA-37446. For Figure A and B clinically relevant haploinsufficient and triplosensitive regions and genes were annotated using the ClinGen database. The red and blue color on in the dosage sensitivity map indicates deletions and duplications respectively. The gray color indicates that there is only moderate indication that the region/gene might be dosage sensitive. Note that *COMT* is overlapped by the 22q deletion, but is not illustrated because it is not part of the ClinGen annotation database.

A

ABCB11 somatic duplication (chr2: 169823286-169851396)
Length: 28Kb, CF: 19.6%

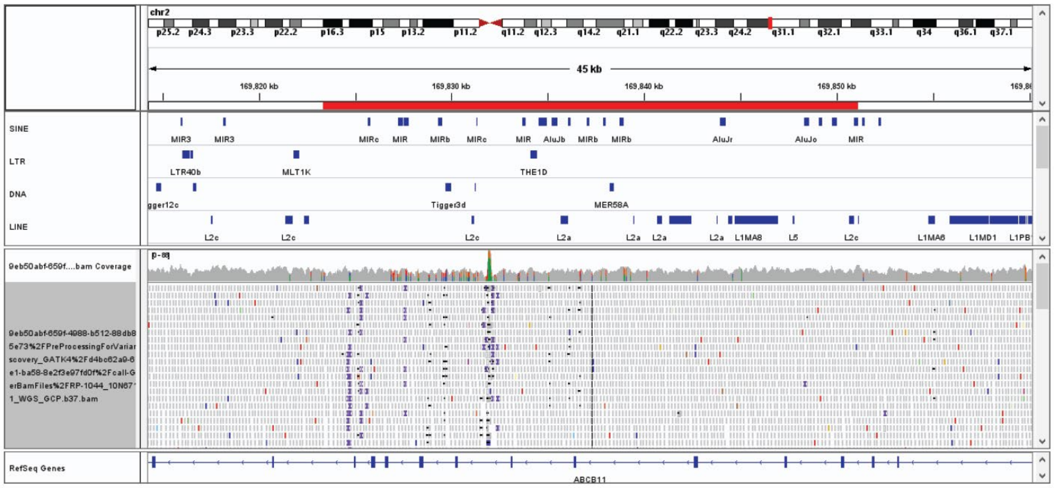

B

ABCB11 somatic deletion (chr2: 169803674-169839081)
Length: 35Kb, CF: 19.1%

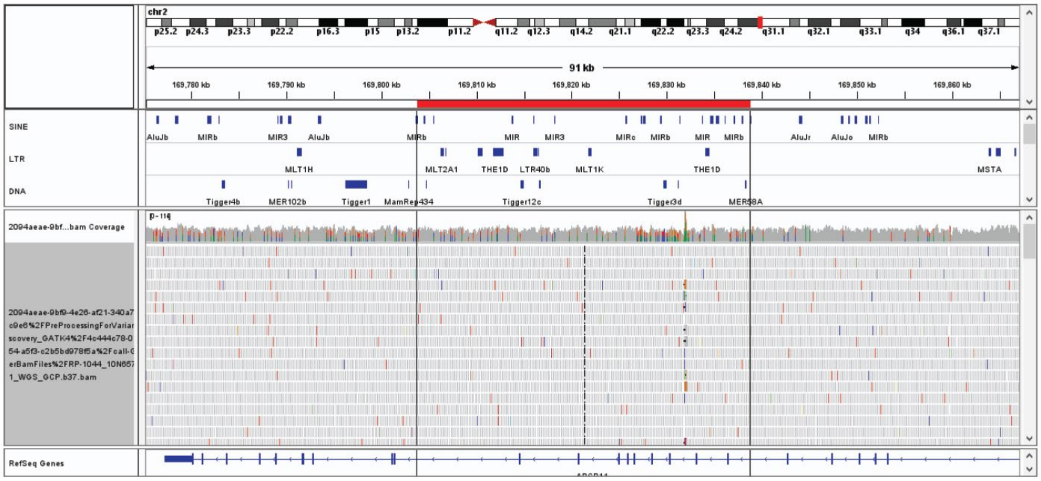

Figure S4: WGS IGV plots of *ABCB11* sCNV samples, related to Figure 4. A, B) IGV plots of *ABCB11* locus. Red bar representing the corresponding putative sCNV region. The tracks from top to bottom on each panel indicates the RepeatMasker annotation for different transposon families, coverage, and reads mapping to that region respectively.

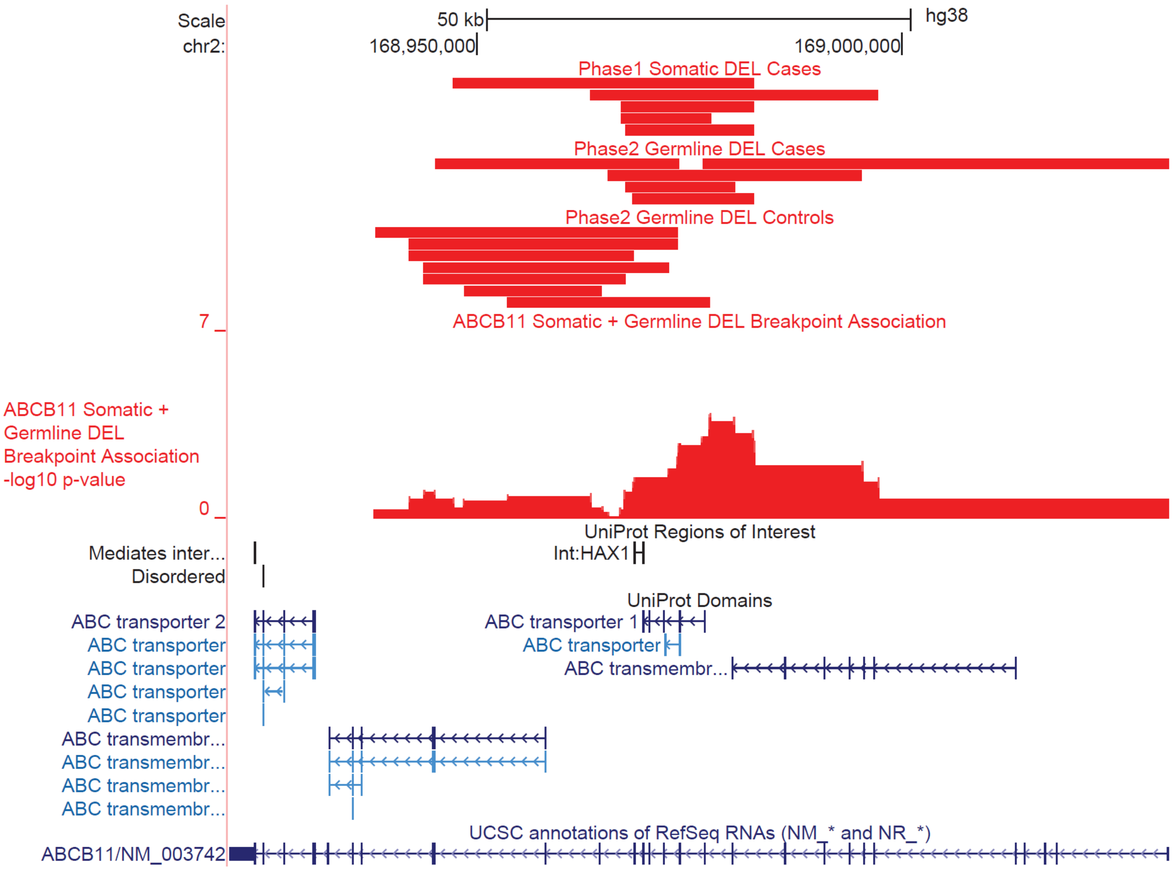

Figure S5: Somatic and Germline deletions of *ABCB11*, related to Figure 4. Adapted GenomeBrowser plot at the *ABCB11* gene locus. Association p-values were computed with logistic regression on disease status, controlling for overall CNV burden.

Table S1: sCNV burden in SCZ cases and controls by gains and losses, related to Figure 1.

Diagnosis	Total Samples	Samples w/ sCNV (#events)	% Occurrence	Samples w/ Gains (# events)	Samples w/ Loss (# events)
Schizophrenia	12,834	118 (131)	0.91	42 (48)	77 (83)
Control	11,648	60 (67)	0.52	22 (22)	39 (45)
Total	24,482	177 (198)	0.72	64 (70)	116 (127)
