# Supplementary material for: Schizophrenia-associated somatic copy-number variants from 12,834 cases reveal recurrent NRXN1 and ABCB11 disruptions
Source: Cell Genom. 2023 Jul 6;3(8):100356. doi: 10.1016/j.xgen.2023.100356 (PMC10435376; doi:10.1016/j.xgen.2023.100356)
Supplement: Document S2. Article plus supplemental information [file mmc3.pdf]

Cell Genomics

Article

Schizophrenia-associated somatic copy-number variants from 12,834 cases reveal recurrent *NRXN1* and *ABCB11* disruptions

Graphical abstract

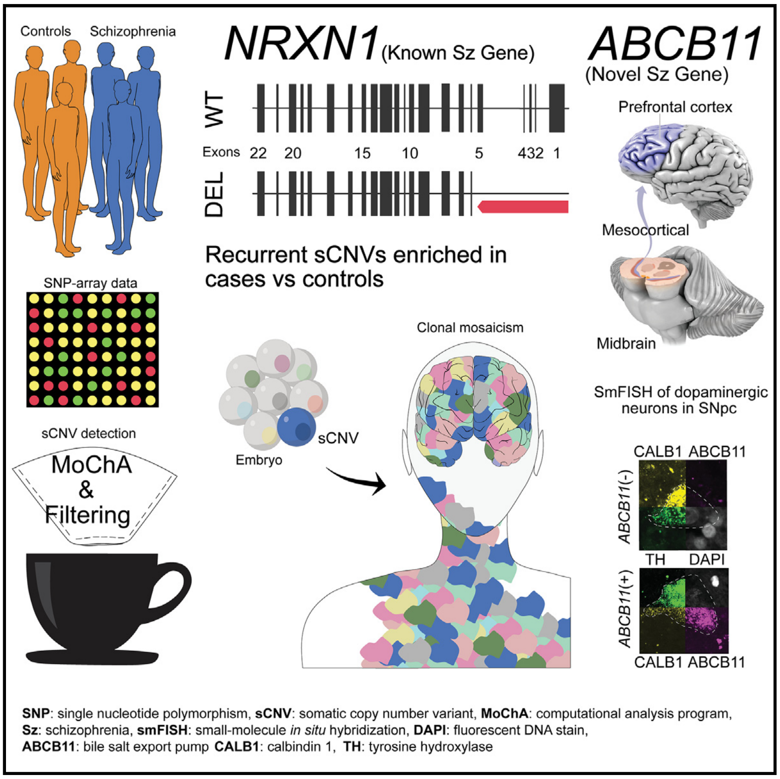

Highlights

- Somatic copy-number variants are more common in SCZ cases than in controls
- Recurrent somatic deletions of *NRXN1* exons 1–5 in SCZ cases
- Recurrent intragenic deletions of *ABCB11* in SCZ cases
- *ABCB11* is specifically enriched in a subset of dopaminergic neurons in human brain

Authors

Eduardo A. Maury, Maxwell A. Sherman, Giulio Genovese, ..., Jonathan Sebat, Eunjung A. Lee, Christopher A. Walsh

Correspondence

christopher.walsh@childrens.harvard.edu

In brief

Maury et al. leveraged blood-derived SNP-array data across 12,834 schizophrenia cases and 11,648 controls to explore somatic copy-number variants (sCNVs). They found higher early-developmental sCNV incidence in cases compared with controls, along with specific intragenic events in *NRXN1* and *ABCB11* that could potentially contribute to SCZ disease.

Maury et al., 2023, Cell Genomics *3*, 100356
August 9, 2023 © 2023 The Author(s).
https://doi.org/10.1016/j.xgen.2023.100356

CellPress

Cell Genomics

CellPress
OPEN ACCESS

Article

Schizophrenia-associated somatic copy-number variants from 12,834 cases reveal recurrent *NRXN1* and *ABCB11* disruptions

Eduardo A. Maury,[1,2,3] Maxwell A. Sherman,[4] Giulio Genovese,[3,5,6] Thomas G. Gilgenast,[7] Tushar Kamath,[6,8] S.J. Burris,[6] Prashanth Rajarajan,[9] Erin Flaherty,[9] Schahram Akbarian,[9] Andrew Chess,[9] Steven A. McCarroll,[3,5] Po-Ru Loh,[3,4] Jennifer E. Phillips-Cremins,[7] Kristen J. Brennand,[9,10] Evan Z. Macosko,[6,11] James T.R. Walters,[12] Michael O'Donovan,[12] Patrick Sullivan,[13] Psychiatric Genomic Consortium Schizophrenia and CNV workgroup[16], Brain Somatic Mosaicism Network[16] Jonathan Sebat,[14] Eunjung A. Lee,[1,3] and Christopher A. Walsh[1,3,15,17,*]

[1]Division of Genetics and Genomics, Manton Center for Orphan Disease, Boston Children's Hospital, Boston, MA, USA
[2]Bioinformatics & Integrative Genomics Program and Harvard/MIT MD-PHD Program, Harvard Medical School, Boston, MA, USA
[3]Program in Medical and Population Genetics, Broad Institute of MIT and Harvard, Cambridge, MA, USA
[4]Brigham and Women's Hospital, Division of Genetics & Center for Data Sciences, Boston, MA, USA
[5]Department of Genetics, Harvard Medical School, Boston, MA, USA
[6]Stanley Center for Psychiatric Research, Broad Institute of MIT and Harvard, Cambridge, MA, USA
[7]Department of Bioengineering, University of Pennsylvania, Philadelphia, PA, USA
[8]Harvard Graduate Program in Biophysics, Harvard University, Cambridge, MA, USA
[9]Nash Family Department of Neuroscience, Friedman Brain Institute, Department of Genetics & Genomics, Icahn Institute of Genomics and Multiscale Biology, Department of Psychiatry, Pamela Sklar Division of Psychiatric Genomics, Icahn School of Medicine of Mount Sinai, New York, NY, USA
[10]Departments of Psychiatry and Genetics, Yale School of Medicine, New Haven, CT, USA
[11]Massachusetts General Hospital, Department of Psychiatry, Boston, MA, USA
[12]MRC Centre for Neuropsychiatric Genetics and Genomics, Division of Psychiatry and Clinical Neurosciences, Cardiff University, Cardiff, Wales
[13]Department of Genetics, University of North Carolina at Chapel Hill, Chapel Hill, NC, USA
[14]University of California San Diego, Department of Psychiatry, Department of Cellular & Molecular Medicine, Beyster Center of Psychiatric Genomics, San Diego, CA, USA
[15]Howard Hughes Medical Institute, Boston Children's Hospital, Boston, MA, USA
[16]Further details can be found in the supplemental information
[17]Lead contact
*Correspondence: christopher.walsh@childrens.harvard.edu
https://doi.org/10.1016/j.xgen.2023.100356

SUMMARY

While germline copy-number variants (CNVs) contribute to schizophrenia (SCZ) risk, the contribution of somatic CNVs (sCNVs)—present in some but not all cells—remains unknown. We identified sCNVs using blood-derived genotype arrays from 12,834 SCZ cases and 11,648 controls, filtering sCNVs at loci recurrently mutated in clonal blood disorders. Likely early-developmental sCNVs were more common in cases (0.91%) than controls (0.51%, p = 2.68e−4), with recurrent somatic deletions of exons 1–5 of the *NRXN1* gene in five SCZ cases. Hi-C maps revealed ectopic, allele-specific loops forming between a potential cryptic promoter and non-coding *cis*-regulatory elements upon 5′ deletions in *NRXN1*. We also observed recurrent intragenic deletions of *ABCB11*, encoding a transporter implicated in anti-psychotic response, in five treatment-resistant SCZ cases and showed that *ABCB11* is specifically enriched in neurons forming mesocortical and mesolimbic dopaminergic projections. Our results indicate potential roles of sCNVs in SCZ risk.

INTRODUCTION

De novo and rare germline copy-number variants (gCNVs) contribute to up to 5.1%–5.5% of schizophrenia (SCZ) cases, with relatively large effect sizes.[1] These gCNVs are usually inherited or represent *de novo* events thought to arise during gametogenesis. Most gCNVs involve several genes, making it difficult to pinpoint specific causative genes. A notable exception is deletion of *NRXN1*, which encodes a presynaptic adhesion protein and has been suggested to have a role in SCZ along with other synaptic genes.[2]

Somatic copy-number variants (sCNVs) present in only a fraction of cells in the body, are increasingly implicated in neuropsychiatric disease.[3–8] For example, a recurrent, large sCNV of chromosome 1q has been repeatedly observed in focal epileptic brain malformations,[9–11] while blood samples from autism

This is an open access article under the CC BY license (http://creativecommons.org/licenses/by/4.0/).

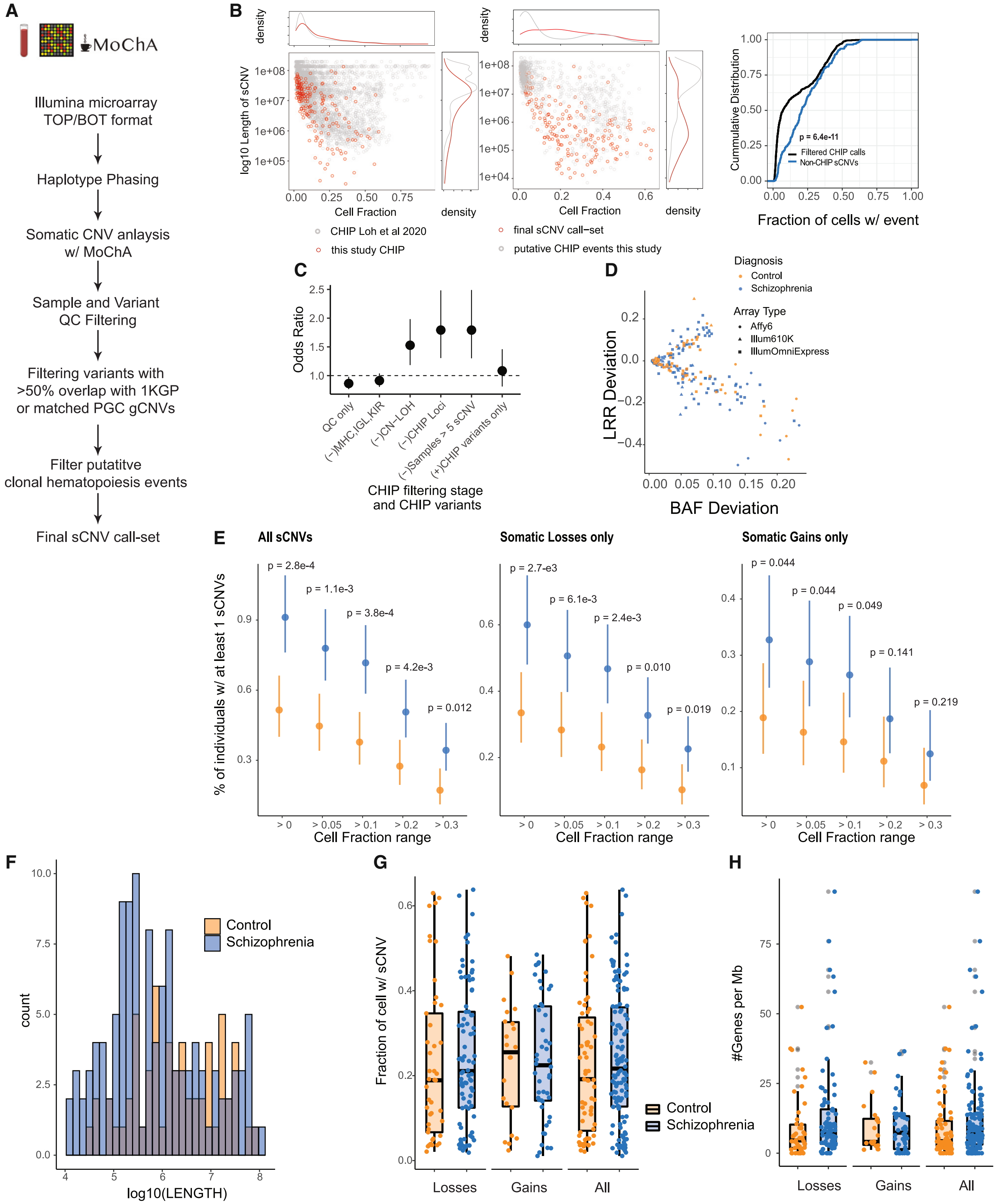

(legend on next page)

spectrum disorder (ASD)[3] showed enrichment of large (>4 Mb) sCNVs, with sCNV size positively correlated with phenotypic severity. The overlap in the genetic architecture of ASD and SCZ[12] suggests the hypothesis that sCNVs may have similar roles in SCZ liability.

Since sCNVs are less common than germline gCNVs, large datasets must be analyzed to assess their contribution to disease, but such large genotyping datasets are generally only available from blood-derived single-nucleotide polymorphism (SNP)-array data created for genome-wide association studies (GWASs), which creates two challenges. The first challenge is that these arrays only capture the earliest developmental events, present in a relatively large fraction of cells[13,14] and hence also likely to be shared in brain cells and other tissues. Prior studies have shown that non-oncological somatic variants present in more than ∼1%–3% of cells in a tissue are typically shared in all developmental lineages in a mosaic fashion.[14–16] The mosaic fraction of variants in blood exhibited a linear relationship with the mosaic fraction in other tissues,[17] suggesting that studying highly mosaic variants in blood might reflect, to an extent, somatic variation in other tissues such as brain.

The second challenge in assessing sCNVs in blood is the increasing recognition that aging and environmental exposures are correlated with sCNVs that are restricted to blood, which are associated with leukemia or pre-cancerous conditions such as clonal hematopoiesis of indeterminate potential (CHIP).[5,18,19] However, CHIP-related sCNVs have now been extensively characterized in dozens of studies in terms of size and mosaic fraction and found to occur at recurrent chromosomal locations that disrupt specific driver genes,[18–22] allowing sCNVs at these loci to be filtered to identify non-CHIP, early-developmental sCNVs that may be associated with SCZ.

In this study, we analyzed SNP-array data from 12,834 cases and 11,648 controls from the Psychiatric Genomic Consortium (PGC) SCZ cohort using a widely utilized, highly sensitive algorithm that leverages haplotype information to detect sCNVs in blood.[3,18,19] We additionally used recent knowledge of the genomic loci of blood events[22] to rigorously filter candidate variants that likely originated from CHIP. We observed an excess of non-CHIP-related sCNVs in SCZ compared with controls and discovered recurrent sCNVs, including recurrent *NRXN1* somatic deletions of exons 1–5 and recurrent intragenic events at *ABCB11* gene as well. Taken together, these data suggest that potential roles of sCNVs in the genetic architecture of SCZ merit further study.

RESULTS

Potential enrichment of non-CHIP sCNV in SCZ cases

sCNVs were identified using the MoChA[18,19] software on 26,186 blood-derived SNP arrays from the PGC2 SCZ cohort[23] (Figure 1A). We removed gCNVs previously identified in subjects of this cohort.[23] Samples that showed signs of contamination, or sCNVs whose copy-number state was not confidently determined, were excluded (STAR Methods). This quality control (QC) led to the identification of 1,341 candidate sCNV, including many presumably related to CHIP, and a subset that may potentially be associated with SCZ.

We identified 1,143 events likely to have arisen from CHIP, based on their chromosomal location at recurrent CHIP regions and resemblance to known CHIP events. We used these CHIP events, which typically have low cell fraction,[18,22] to compare the performance of MoChA in our dataset with prior studies. The events identified as CHIP in our initial call set followed a similar distribution of cell fraction (CF) and length compared with well-known CHIP events from the UK Biobank[19,22] (Figure 1B, left panel). This similarity suggests that our pipeline identifies sCNVs in varied patient datasets with high confidence.

While there is variation across cohorts for the number of CHIP events identified, on average the rates of CHIP events were similar in cases compared with controls. Pooling all the CHIP events did not show a significant difference in CHIP events in SCZ compared with controls (Fisher's exact test odds ratio [OR], 1.08; 95% confidence interval [CI] [0.81–1.46], $p = 0.618$; Figure 1C). Performing meta-analysis to account for potential batch heterogeneity, similarly, revealed no significant decrease in CHIP events across cases and controls (one-sided Fisher's exact test, Liptak's combined p value = 0.9; Figure S1A). The mean number of CHIP events across cohorts for SCZ samples was 0.046 (SE = 0.009), similar for controls with 0.041 (SE = 0.008). Since we do not have age information on all samples, it is possible that any difference in CHIP burden in SCZ and controls might be masked by differential age distribution or other environmental factors. Nevertheless, this result suggests similar sCNV detection sensitivity in cases compared with controls in our dataset.

We next filtered likely CHIP-related events and identified a subset of early-developmental sCNVs, most present in a high CF. Specifically, we removed all copy-neutral loss of heterozygosity (CN-LOH), loci commonly altered in the immune system (e.g., major histocompatibility locus [MHC]) and other known common

Figure 1. Somatic CNV burden in SCZ

(A) Schematic of sCNV calling and filtering.

(B) Left: scatterplot and marginal distributions of length and CF of sCNVs identified as CHIP vs. non-CHIP. Middle: distribution of canonical CHIP events in sCNVs identified as CHIP in our call set compared with CHIP events identified in the UK Biobank.[19] Right: cumulative distributions of CF of CHIP vs. non-CHIP events; p value from Kolmologorov-Smirnov test.

(C) Odds ratio plots comparing sCNV burden across different CHIP filtering stages. Odds ratios and 95% CI were derived from Fisher's exact test. CHIP variants were defined as those overlapping canonical CHIP events.[22]

(D) Trident plot of final call set. Each point represents an event, with colors and shapes indicating subject's diagnoses and array type.

(E) Percentage of individuals with ≥1 sCNV in cases and controls across different minimum CF thresholds. Dots represent mean fraction and lines represent 95% CI from the binomial distribution using Wilson's score interval with Newcombe modification; p values calculated with two-sided Fisher's exact test.

(F) Histogram of sCNV size (log10 scale) in cases and controls.

(G) Boxplots of sCNV CFs in cases vs. controls.

(H) Boxplots of the number genes per megabase of sCNVs in cases and controls.

CHIP loci[19,21] and filtered outlier samples with multiple events (>5 sCNVs) (Figure 1A) (STAR Methods). Our non-CHIP events show a different distribution of CF and length compared with events identified as CHIP (Figure 1B, middle panel). sCNVs that occur early in development are clonally shared across multiple tissues and are thus expected to be present at larger CF than those occurring through CHIP alone. Reassuringly, variants filtered as potential CHIP exhibited significantly lower CF than non-CHIP events (Wilcoxon Rank-Sum test p = 6.4e−11) (Figure 1B). This difference suggests that our filtering reliably removes likely CHIP events, although some *bona fide* early-developmental sCNVs may be filtered out as well, especially those coming from CN-LOH events. While we found equivalent rates of CHIP in SCZ samples and controls (Figure 1C), stepwise removal of likely CHIP variants showed increasing enrichment of the remaining sCNVs in SCZ compared with controls, with the highest effect size once all the CHIP-related events were removed (Figure 1C).

While non-recurrent or small events may be difficult to detect, Loh et al.[18] demonstrated detection sensitivity for events as small as 100 kb. There is an inverse-square relationship between event size and CF, such that, at a CF of ∼10%, events > 1 Mb are detectable, while sCNVs > 100 Mb need to be present in ∼1% of cells to be detected.[18] Since CHIP events tend to occur at lower CF,[18,19,21,22] consequently MoChA would have sensitivity to detect only larger events. On the other hand, it is expected from the modeling of MoChA[18] and the inverse-square relationship that early-developmental events, such as those predicted to have occurred in some individuals in our study, have a biological higher CF, and hence that MoChA should have sensitivity to detect them even if they are smaller.

Whole-genome sequencing supports presence of sCNV

We confirmed several likely non-CHIP sCNV using 40–60× whole-genome sequencing (WGS) in five individuals. We detected a probable 650-kb somatic deletion in one individual with a predicted CF of 52%, which was supported by simple inspection of the Integrative Genomics Viewer (IGV) read pileup (Figure S2). The CF estimated from WGS was ∼50%, closely matching the MoChA estimate. We also called a large 26-Mb somatic deletion in 9q21.11-9q22.2 (hg19 coordinates, chr9:71033538-97246817) with an estimated CF of 43% in another individual. Running MoChA on WGS also supported a similar somatic deletion overlapping the original event in the same individual (hg19 coordinates chr9, 38767760–97259994) with a CF of 46% and with a size larger than seen on the SNP array, attributable to the event extending into the centromeric region that is not well represented on SNP arrays.[24] WGS also confirmed both CFs and breakpoints for three of three much smaller *NRXN1* deletions presented in detail below. Since the data for this study was generated across different countries, precluding access to DNA for more widespread validation of MoChA calls, we applied MoChA conservatively, only calling variants (for both cases and controls) in a range of size and CFs that have been shown in prior papers with this algorithm to have a negligible false-discovery rate.[3,18–20,25] However, further studies will be required to provide more precise estimates of prevalence of sCNV in SCZ, and rate comparisons in our study should be interpreted with caution.

Analysis of putative early sCNVs in SCZ and controls

sCNVs not related to CHIP occurred in a small but significant fraction of SCZ cases. From the initial 13,464 SCZ cases and 12,722 controls, a total of 12,834 cases and 11,648 controls remained after QC. The final non-CHIP sCNV call set consisted of 198 events in 178 individuals, made up of 127 losses and 70 gains (Tables S1and S2; and Figure 1D). These events ranged in CF from 1.10% to 63.8% (median, 21.1%), and ranged in size from 10.7 kb to 95.3 Mb (median, 686.0 kb). The high CF of events in samples without a blood cancer diagnosis suggests that these somatic variants might have arisen during early-developmental stages.[13,14,17] The percentage of individuals with at least one sCNV was 0.91% in SCZ and 0.51% in controls (OR, 1.78; 95% CI, 1.29–2.47; two-sided Fisher's exact test, p = 2.68e−4) (Figure 1E). The sCNV incidence in controls was comparable with unaffected siblings in an earlier study (0.51% vs. 0.54%),[3] while our estimates in SCZ were higher compared with the ASD cases from the same study (0.91% vs. 0.58%).[3] This higher rate most likely reflects sensitivity improvement in the pipeline since the earlier study, although we cannot rule out differential sources of artifact or biological effect (STAR Methods). Prior analyses with MoChA have estimated burdens of sCNV (with CF > 10%) in blood samples of individuals with no history of hematologic cancer of 3.2% in the UK Biobank, 5.2% in the Mass General Brigham Biobank, 5.9% in FinnGen, and 1.3% in Biobank Japan.[20] These numbers are all larger than what we observe in our SCZ cohort and likely reflect our filtering of CHIP variants and/or differential environmental exposures.

To rule out potential residual CHIP events in our call set contributing to the difference in prevalence of sCNVs, we performed the burden test using different minimum CF cutoffs, with higher CF cutoff being less likely to be CHIP events and more likely to be early-developmental events. There remained a statistically significant enrichment in SCZ through several ranges, even when events were split into losses and gains (Figure 1E). We further accounted for potential batch heterogeneity (Figure S1A) using meta-analysis across each study batch containing both cases and controls, obtaining a Liptak's combined p value of 0.032 using a one-sided Fisher's exact test. To further confirm that the enrichment observed was not driven by CHIP events, we removed all variants from samples with suspected CHIP variants (30 variants removed). With this smaller call set, we still obtained a significant enrichment in SCZ cases for sCNV compared with controls (Fisher's exact test, OR, 1.64; 95% CI [1.16:2.33]; p = 0.0041).

In contrast with previous findings in ASD,[3] sCNVs in SCZ cases were of similar size compared with control after accounting for different arrays/cohorts by mixed-effect modeling (p = 0.26) (Figure 1F). These events were also present at similar CF in cases compared with controls (p = 0.986; Figure 1G). There was also no detectable difference in gene density (p = 0.08; Figure 1H). These trends were observed across the different batches as well (Figure S1B–D). In contrast to gCNV,[1,23] sCNV did not show overall gene-set enrichment for the top 20% expressed brain genes (p = 0.14), synaptic genes (p = 0.12), or haploinsufficient genes as measured by a probability of of being loss-of-function intolerant (pLI) score >0.90[26] (p = 0.54). We

CellPress
OPEN ACCESS

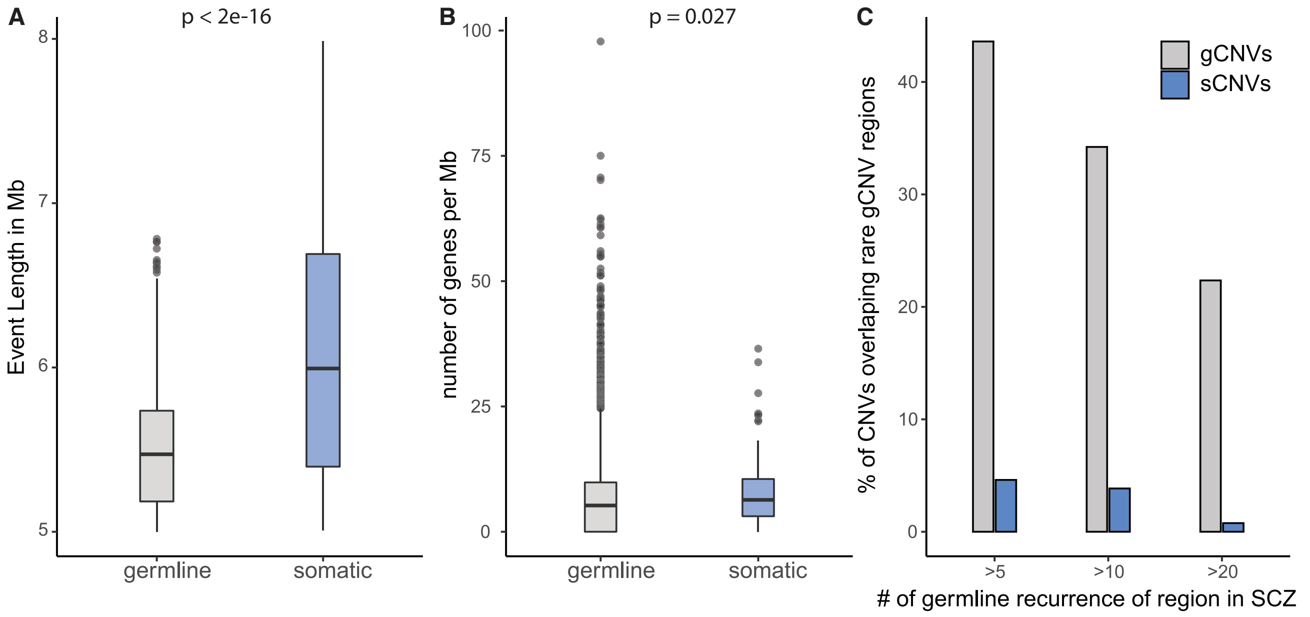

Figure 2. Somatic CNVs differ in size, gene content, and location from gCNVs in SCZ

(A) Boxplot of event length in SCZ in somatic and germline state.

(B) Plot of number of genes affected per megabase; p values for (A) and (B) were calculated using mixed-effect model log-normal and negative binomial regression, respectively, with batch as a random effect.

(C) Bar plots showing percentage of CNVs in each category that overlapped recurrent germline rare CNV regions in SCZ across three different minimum recurrence thresholds.

did not detect events in the top 10 genes related to SCZ by the SCHEMA consortium[27] or the presence of two-hit events (germline + sCNVs) in our dataset.

Some sCNV overlapped cytobands previously implicated in SCZ but showed distinctive features. While one SCZ case had a 4.1-Mb somatic deletion in cytoband 16p11.2, it was not only significantly larger than the canonical germline 16p11.2 deletions (<600 kb) observed in SCZ and ASD[23,28] but also the mosaic deletion did not overlap the canonical proximal or distal events (Figure S3A). We also observed one SCZ case with a somatic deletion in the 22q11.21 locus that was significantly smaller (686 kb) than the recurrent germline 22q11.21 deletions observed in SCZ (2.35 Mb) (Figure S3B). The mosaic 22q11 deletion we observed, however, overlapped the genes *TBX1* and *COMT*, which have been suggested as key genes driving some of the phenotypic effects and SCZ risk of germline 22q11 deletion.[29,30]

Predicted sCNV are larger and affect more gene-dense regions compared with gCNVs

Comparison of the genomic features of sCNVs with rare (minor population allele frequency <0.5%) gCNVs calls of SCZ cases from the arrays used in our current study[23] showed that sCNVs were larger (fold change, 4.57; 95% CI, 3.76–5.48; mixed-effect log-normal regression $p < 2e-16$) and involved more genes (fold change, 1.27; 95% CI, 1.03–2.56; mixed-effect negative binomial regression $p = 0.027$) (Figures 2A and 2B). We observed that genomic regions affected by rare gCNVs present in at least five SCZ cases overlapped 43.6% of all the gCNVs, whereas these same regions overlapped only 4.48% of SCZ sCNVs (Figure 2C). This difference in genomic regions persisted throughout for rare gCNVs present at different minimum recurrence cutoffs (Figure 2C). These findings suggest that, with sufficient statistical power, mosaic events might offer additional new insights into different risk regions of the genome.

Recurrent, intragenic deletions in *NRXN1* observed in SCZ

Six individuals showed somatic deletions in cytoband 2p16.3 affecting only the *NRXN1* gene, at remarkably stereotyped and distinctive regions of the gene. The size of these events ranged from 105 to 534 kb, with CF ranging from 13.8% to 43.1%, suggesting that they occurred early in development. One deletion was limited to intron 5 (Figure 3A) and is of uncertain disease significance since multiple germline deletions of this intron have been reported in control individuals.[23] In contrast, the remaining five 2p16.3 deletions consistently removed exons 1–5 of *NRXN1*α while leaving exon 6 and the rest of the gene intact. This stereotyped five-exon deletion contrasts with germline deletions in *NRXN1*, previously implicated in SCZ,[23,31] which show highly variable breakpoints and relationships to *NRXN1* exons.[23,32,33] Therefore, the recurrent, mosaic deletion of the same exons 1–5 in all five exonic deletions would seem to demand a specific mechanistic explanation. To further assess the prevalence of somatic *NRXN1* deletions, we re-ran MoChA with a more lenient threshold and checked whether *NRXN1* copy-number variants (CNVs) identified in the original PGC study[23] as germline might in fact be somatic. This strategy revealed an *NRXN1* deletion previously identified as germline,

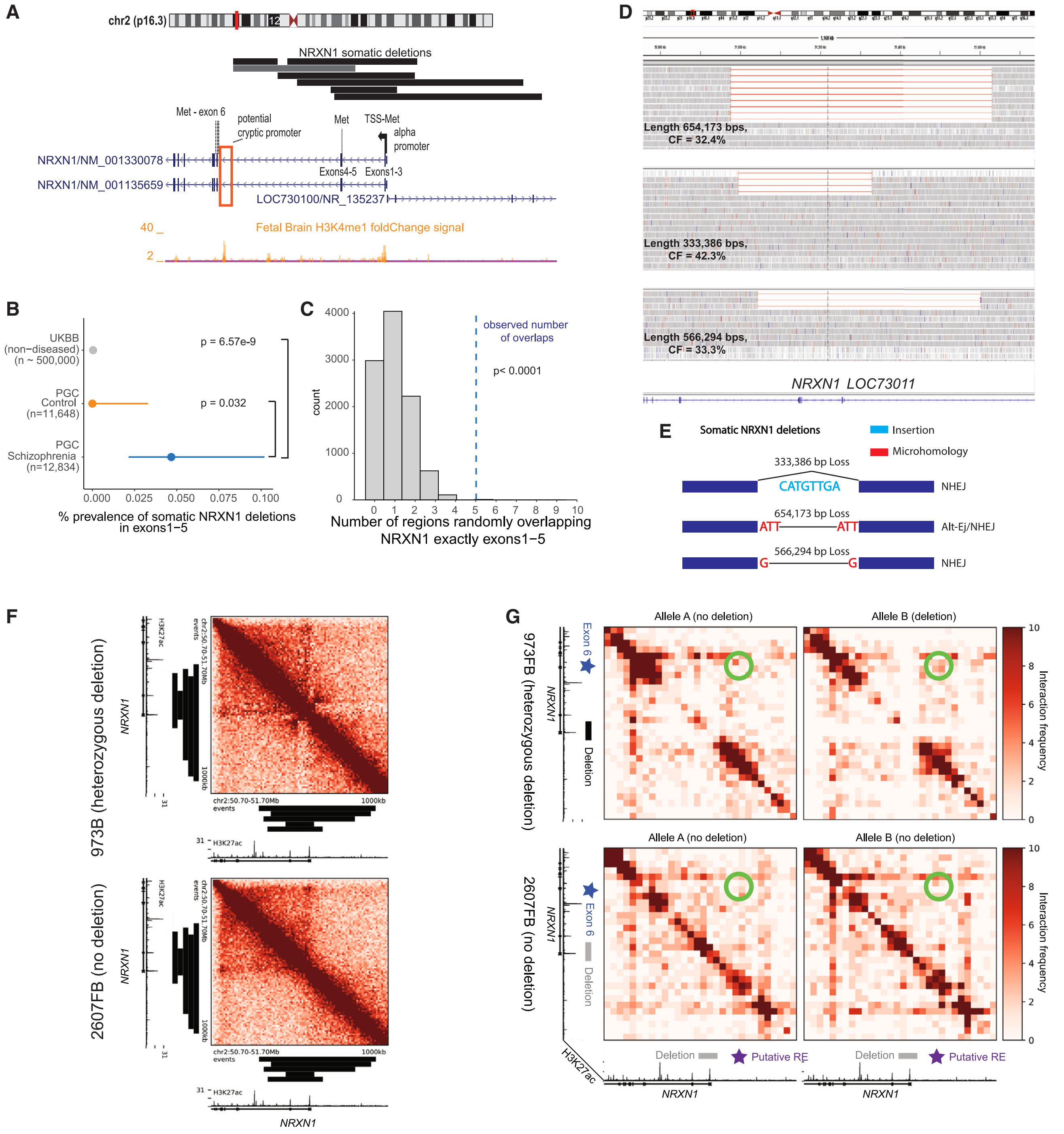

Figure 3. Somatic deletions of *NRXN1* exons 1–5

(A) Adapted GenomeBrowser view of seven somatic deletions of *NRXN1*. The alpha promoter and in-frame ATG/methionine sites on exons are annotated for *NRXN1*. Histone marks were obtained from Roadmap epigenomics tracks.[34] Potential cryptic promoter/enhancer is marked by a red box. Gray horizontal bar indicates CNV previously called germline that was found to be somatic.

(B) Prevalence of somatic deletions of *NRXN1* exons 1–5 in SCZ, controls, and UK Biobank; p values were estimated using two-sided Fisher's exact test, and 95% CIs were obtained using the Wilson's score interval with Newcombe modification.

(C) Histogram of the distribution of number of overlaps of NRXN1 exons 1-5 from randomly shuffling the discovered NRXN1 sCNVs across the NRXN1 locus. The blue dashed line is the observed number of overlaps, which is equal to six.

(D) IGV plots of the deletions of three SCZ subjects with somatic deletions in *NRXN1* exons 1–5 from WGS. For clarity, not all the reads are shown.

(legend continued on next page)

with an estimated CF of 41%, consistent with being somatic. This variant appeared to overlap exons 4–5 for *NRXN1* (Figure 3A), although its exact boundaries are uncertain.

Comparing the burden of *NRXN1* somatic deletions in SCZ cases vs. controls revealed significant enrichment in cases (two-sided Fisher's exact test p = 0.032, exonic only; p = 0.016, exonic + intronic; Figure 3B). Previously generated sCNV calls from the UK Biobank[18,19] identified two persons without history of psychiatric disorder (of ~500,000 individuals) with similar sCNV breakpoints affecting exons 1–5 in *NRXN1*. Although the arrays used in the UK Biobank have different sensitivity compared with arrays used in this study, they should have comparable sensitivity to detect these large events at CF > 10%.[18] Consequently, while we cannot fully rule out batch effect bias, combining our results with the UK Biobank suggest an enrichment of exon 1–5 *NRXN1* deletions in the somatic state in SCZ samples (OR, 117.08; 95% CI, 20.91–1165.84; Fisher's exact test p = 6.57e−9; Figure 3B). To further assess whether we could have observed five overlaps of exons 1–5 by chance, we performed a bootstrap test by randomly shuffling regions of length equal to the seven *NRXN1* sCNV that we discovered across the *NRXN1* locus and computed the number of random overlaps with exons 1–5. We observed that five random overlaps of exactly exons 1–5 was a highly unlikely event (p < 0.0001; see STAR Methods; Figure 3C). A similar study with a similar pipeline and dataset to this study[3] on ASD and control samples did not detect somatic deletions in *NRXN1* overlapping exons 1–5, suggesting relative specificity of this event to SCZ.

We were able to obtain 40× WGS from three *NRXN1α* deletion cases processed at the Broad Institute, confirming that each event removed exons 1–5 of the gene with estimated CFs of 42.4%, 33.3%, and 32.4%, as expected (Figure 3D), and defining their breakpoints at base-pair resolution. WGS analysis showed that none of the exact *NRXN1* sCNVs breakpoints were recurrent or overlapped known interspersed repeats or low-complexity DNA sequences, which could have predisposed this genomic region to genomic instability.

Further breakpoint analysis of these *NRXN1* sCNVs using previously established classification criteria[35,36] (Figure 3E) suggested diverse mechanisms of formation. One event had only 1 bp of microhomology (MH), suggesting that this event arose via non-homologous end-joining repair (NHEJ). Another event had a 3-bp MH, implicating an alternative end-joining repair mechanism (alt-EJ). The last event had no MH but revealed 8 bp inserted at the breakpoint, small enough to have occurred by non-template directed repair associated with NHEJ, although it is also possible that a fork-stalling template switching mechanism might have occurred,[37] but this mechanism tends to produce insertions >10 bp and usually occurs where some microhomology exists.[35] Taken together, these results suggest that the somatic deletions of *NRXN1* that we observed do not disrupt recurrent exons due to instability of the genomic region around the events.

NRXN1 deletions suggest a potential cryptic promoter in human induced neurons

The absence of a genomic mechanism for the recurrent somatic deletions in *NRXN1α* suggests an alternative hypothesis, that the recurrence reflects an unknown but specific effect of these deletions on *NRXN1* gene function. These sCNVs overlap the *NRXN1α* promoter and the first in-frame ATG transcription start site, which would be expected to disrupt transcription of the full alpha isoform (Figure 3A) while leaving downstream beta and gamma isoforms intact. Intriguingly, the somatic deletions leave intact H3K4Me1 histone marks that lie just 5′ from exon 6, which contains an in-frame ATG (Figure 3A). These features might indicate a cryptic promoter or enhancer adjacent to the in-frame ATG in exon 6, potentially producing an N-terminal truncated *NRXN1α*. This truncated protein would lack the signal peptide required for shuttling to the cell surface, potentially causing abnormal trafficking. Similar germline *NRXN1* deletions have been shown to cause accumulation of the NRXN1 intracellular binding protein CASK in human induced pluripotent cells (iPSCs) from SCZ patients.[38]

To further explore functional effects of somatic deletions in the 5′ end of *NRXN1*, we generated Hi-C data from neurons differentiated from human iPSCs (hiPSCs) containing heterozygous germline deletions in the 5′ end (exons 1–2) and compared them with an iPSC line that had no germline deletion in *NRXN1* (STAR Methods). Unphased Hi-C heatmaps in iPSC neurons showed that somatic deletions affecting exons 1–5 all fully overlap the topologically associating domain (TAD) boundary co-localized with the alpha promoter (Figure 3F). Recently, disruption of TAD boundaries by germline structural variants have been associated with developmental disorders as well as SCZ.[39,40] These observations together suggest that 5′ *NRXN1* deletions might disrupt the structural integrity of the TAD boundary in SCZ and could result in ectopic enhancer-promoter mis-wiring and dysregulated gene expression.

To investigate possible 3D genome miswiring due to *NRXN1* deletions, we generated allele-specific, phased Hi-C maps in both control as well as deletion-carrying SCZ iPSC-neurons (STAR Methods). Surprisingly, we observed the *de novo* formation of an ectopic looping interaction (Figure 3G, green circle) between exon 6 of *NRXN1* (Figure 3G, blue star) and a putative non-coding *cis*-regulatory element upstream of the *NRXN1* alpha promoter (Figure 3G, purple star). This ectopic loop appeared to be specific to the deletion-harboring allele of the sample bearing a heterozygous deletion spanning the alpha promoter at the 5′ end of *NRXN1* (973FB) and was not observed on either allele in samples that lacked the deletion (2607FB). Because the interaction spans the deleted region, we hypothesize that the deleted region contains an element with some degree of boundary function preventing this loop from forming normally. Consistent with our hypothesis, the frequency of non-specific interactions increased across the boundary only on the *NRXN1*-deleted allele, suggesting

(E) Breakpoint analysis schematic showing observed insertions and microhomology at breakpoints of *NRXN1* sCNVs along with event length. NHEJ, non-homologous end-joining repair; Alt-EJ, alternative end joining.

(F) Unphased Hi-C heatmap for hiPSC-derived neurons with and without 5′ (exon 1and 2) deletions. Black bars indicate regions of somatic *NRXN1* deletions.

(G) Phased Hi-C heatmaps for hiPSC-derived neurons. Green circles indicate areas of higher signal with 5′ deletion of *NRXN1* in the affected allele. Black bar indicates germline *NRXN1* deletion of exons 1and 2. RE, regulatory element.

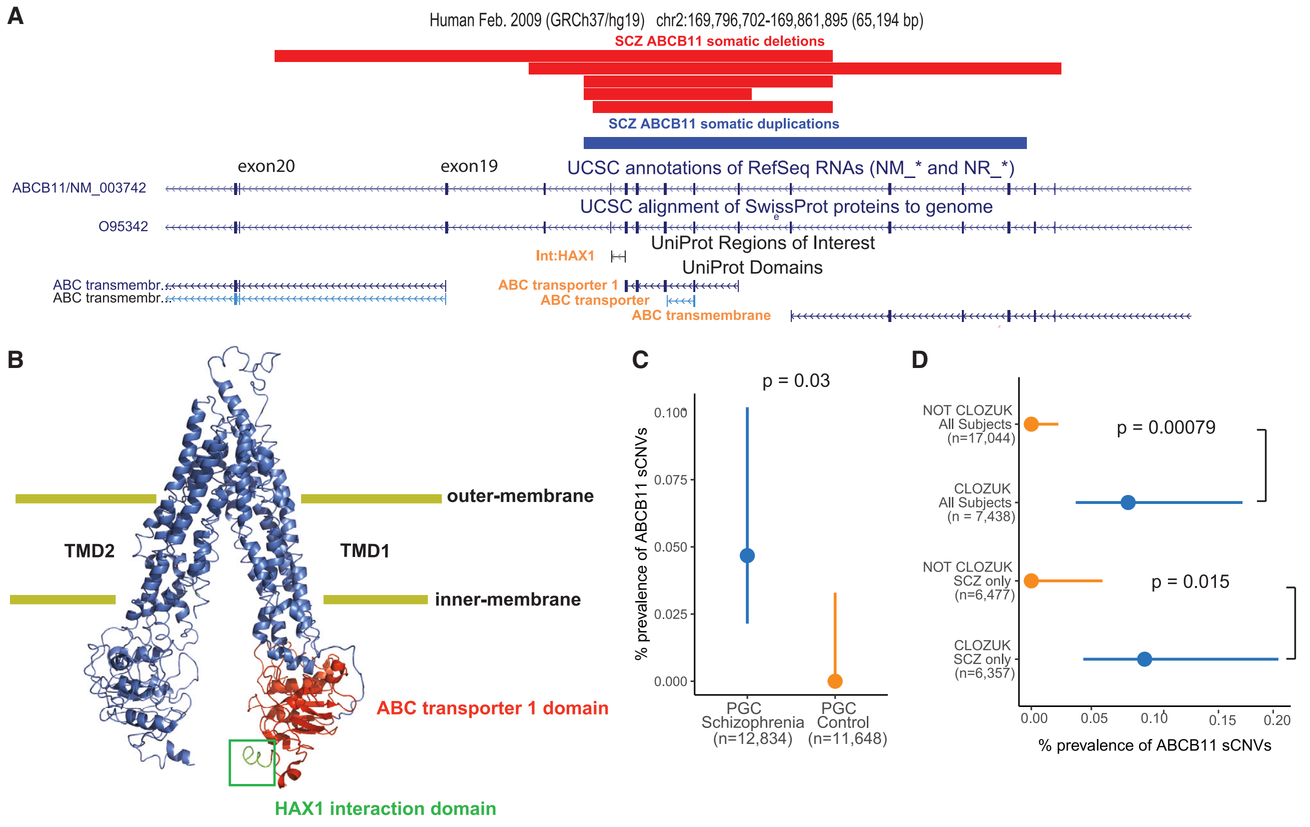

Figure 4. Somatic CNVs in treatment-resistant SCZ subjects overlap the *ABCB11* gene

(A) Adapted GenomeBrowser view of five somatic deletions and one somatic duplication of *ABCB11*. Protein domains of interest overlapped by the sCNVs have orange font.

(B) PyMOL schematic of the ABCB11 protein shows HAX1 protein interaction region and the ABC transporter 1 domain, which are affected by somatic deletions of *ABCB11*. The protein is on an "inner-open" conformation, not bound to ATP.

(C) Prevalence of intragenic sCNV in *ABCB11* in SCZ and controls.

(D) Prevalence of intragenic sCNV in *ABCB11* in CLOZUK cohort samples. For (C) and (D), p values were estimated using two-sided Fisher's exact test, and 95% CIs were obtained using the Wilson's score interval with Newcombe modification.

allele-specific compromise of TAD structural integrity in SCZ (Figure 3G). Together, a working model is that *de novo* looping interaction in 5′ *NRXN1* deletions in SCZ connecting exon 6 to a putatively regulatory element could promote spurious pathological transcripts initiating at exon 6, although other alternative explanations remain as well.

Recurrent sCNVs in the *ABCB11* gene observed in treatment-resistant SCZ cases

We identified six SCZ cases with focal sCNVs within the *ABCB11* gene (five deletions and one gain; Figure 4A), which has previously been associated with anti-psychotic response.[41,42] These sCNVs were all smaller than average, from 10.5 to 35.4 kb, but also with high CFs (18.3%–26.8%), suggesting that they also occurred early in development. *ABCB11* encodes a member of the ATP-binding cassette (ABC) transporter superfamily and has a key role in transporting bile acids across the cell membrane[42] in hepatocytes, the cells involved in a wide range anti-psychotic metabolism. Biallelic loss-of-function variants in *ABCB11* result in severe pediatric-onset liver disease, with many patients developing malignancies or pathological complications within the first decade of life.[43–46] All the *ABCB11* sCNVs overlapped the ABC transporter 1 domain and the domain responsible for interaction with the HAX1 protein (Figure 4B), the latter facilitating internalization of ABCB11 via clathrin-mediated endocytosis.[47,48] Consequently, deletions might not only alter the protein's function by altering the transporter domains but also prevent removal of ABCB11 from the cell surface, perhaps leading to a dominant-negative loss of function. Since the sCNVs in *ABCB11* do not overlap the gene's promoter and there are in-frame ATG sites in downstream exons 19 and 20, a truncated protein could be produced. The consequences of the somatic duplication event are less clear. We also note that four out of five deletions and the duplication overlap one of the transmembrane domains, further supporting the idea that these sCNVs might have a detrimental effect on ABCB11 function. The case-control enrichment of *ABCB11* sCNVs was statistically significant (two-sided Fisher's exact test, p = 0.03; Figure 4C).

All six cases with *ABCB11* sCNV came from batches of CLOZUK,[49] a treatment-resistant SCZ (TRS) cohort. These

CellPress
OPEN ACCESS

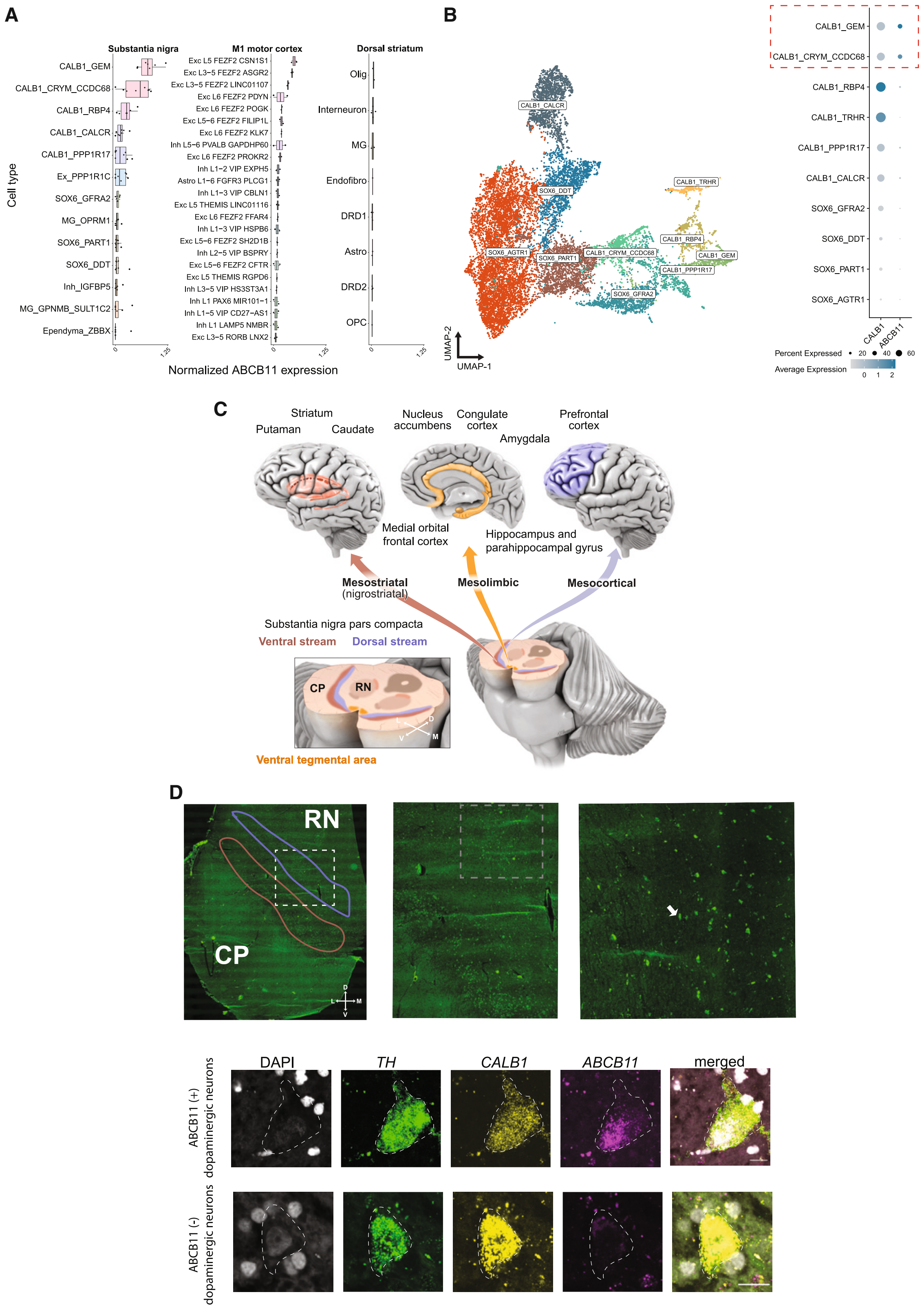

(legend on next page)

samples were obtained from individuals with TRS, taking clozapine, and thus subject to standard blood monitoring for this drug.[50] Even though the CLOZUK samples constituted a significant portion of our study, observing six cases from only this cohort represents a statistically significant enrichment (two-sided Fisher's exact test, p = 0.00079, and p = 0.015 for SCZ only; Figure 4D). *ABCB11* sCNVs were not found in any previous analyses of healthy individuals from the UK Biobank and Biobank Japan.[19,21] Thus, these variants might plausibly regulate either SCZ liability or treatment response. Of the samples with *ABCB11* sCNV, only two (one gain and one loss) were available for WGS. The predicted breakpoints fall in repetitive regions (short interspersed nuclear elements [SINE]) (Figure S4), making it difficult to identify exact breakpoints, although the presence of these repetitive sequences suggests a potential mechanism of somatic deletion through microhomology. It is also possible that this part of the genome is unstable, since the *ABCB11* gene has a significant burden of Alu-family members flanking exons as quantified by the AluAluCNV predictor score of 0.46, potentially implicating Alu-Alu-mediated rearrangement (AAMR).[51]

Combining the *ABCB11* somatic deletions we observed in our SCZ cases with germline deletions identified as part of the phase 2 PGC gCNV dataset revealed robust overlap between the mosaic deletions we detected and those present in separate SCZ cases in the germline state. There were five SCZ cases with gCNVs at the *ABCB11* locus, with three of them coming from the CLOZUK cohort (Figure S5). We were not able to obtain clinical data to determine whether the remaining two cases had TRS. Although six controls showed germline *ABCB11* deletions, these events tended to cluster downstream of the SCZ gCNV and sCNV variants (Figure S4). SCZ risk association analyses combining germline and somatic deletions of *ABCB11* revealed a nominally statistically significant association of sCNV at the HAX1 interaction site and ABC transporter 1 site (peak association, p = 1.4e−4), although this did not meet the threshold (p = 8.3e−8) for genome-wide significance.

ABCB11 is enriched in human dopaminergic neurons residing in the dorsal tier of the substantia nigra pars compacta

While *ABCB11* has been primarily studied in hepatocytes, we explored whether it might show expression in human brain. In publicly available single-nuclei RNA-sequencing data from three brain regions—cortex, caudate nucleus, and substantia nigra pars compacta (SNpc)[52,53]— across the 151 cell types surveyed from these regions, we found that two dopaminergic (DA) populations in the SNpc showed the strongest expression of *ABCB11*, along with expression in subpopulations of layer 5 excitatory neurons in motor cortex (Figure 5A). The two *ABCB11*-expressing substantia nigra (SN) DA populations also showed strong expression of *CALB1* in a recent survey of human midbrain DA neurons[52] (Figure 5B). Interestingly, calbindin-positive DA neurons reside in the dorsal tier of the SNpc, which projects to the ventral striatum, amygdala, as well as to cortical areas through the mesolimbic and, more preferentially, the mesocortical pathways (Figure 5C).[52,54,55] These projections have been repeatedly implicated in SCZ pathology and treatment response.[56]

We validated the expression of *ABCB11* in human DA neurons of the SNpc with single-molecule fluorescence *in situ* hybridization (smFISH) across the midbrain of a postmortem neurotypical control. In support of the small nuclear RNA sequencing (snRNA-seq) data, we found multiple *TH*+ (tyrosine hydroxylase, the gene encoding the rate-limiting enzyme for dopamine production)/ *CALB1*+/*ABCB11*+ cells residing in the midbrain pars compacta region (Figure 5D). We also noted *ABCB11* expression in EXC L5 FEZF2 CSN1S1 and EXC L3-5 FEZF2 ASGR2 cells (Figure 5B), which correspond to Betz cells in the primary motor cortex[53]; however, the relationship of this cell type to SCZ is less clear and we cannot rule out that *ABCB11* might be present in other related excitatory layer 5 neurons.

DISCUSSION

We show that somatic CNVs may contribute a small but significant part of the genetic architecture of SCZ, mirroring previous findings of rare germline and *de novo* CNVs,[1,23] but involving a much more modest proportion of cases. The estimated excess burden of sCNV in SCZ would be 0.4%, which represents a preliminary estimate; we are limited to detecting events with large enough CFs to be present as mosaics in different tissues such as blood and are not able to assess events that might be restricted to brain, in addition to limitations of sequencing validation to better characterize potential sources of artifact. Future studies with additional orthogonal validation, accounting for hereditary stratification and germline background risks, might provide more accurate estimates of the risk carried by sCNVs in SCZ.

In this study, we also report the discovery of five SCZ cases with mosaic deletions of exons 1–5 that also cover the promoter of *NRXN1*α. Deletions of these exons were present in only two

Figure 5. Expression of *ABCB11* in human brain DA neurons

(A) Boxplot of log-normalized *ABCB11* expression across three brain regions. Each point indicates an individual sample. Cell type annotations obtained from Kamath et al. (for SN and dorsal striatum samples) and Bakken et al. (for M1 motor cortex samples). Ex/Exc, excitatory neurons; Inh, inhibitory interneurons; Olig, oligodendrocytes; MG, microglia/macrophages; Endofibro, endothelial cells/pericytes; DRD1, direct spiny projection neurons; DRD2, indirect spiny projection neurons; Astro, astrocytes; OPC, oligodendrocyte precursor cells.

(B) Left: uniform manifold approximation projection (UMAP) of low-dimensional embedding of 15,684 DA neurons from eight neurotypical donors. Points are colored by clusters obtained from Kamath et al.[52] Right: dot plot of normalized *ABCB11* expression across 10 DA subtypes.

(C) Schematic of major DA projections from dorsal and ventral streams of SN pars compacta to cortical areas associated with SCZ.

(D) Top row: tiled image of a postmortem midbrain tissue section with increasing magnification. Right: white dashed box corresponds to approximate location of middle image and similarly, for the middle image, white boxed arrow with the right image. Red outline indicates approximate ventral tier and blue is approximate dorsal tier. Bottom row: representative image of smFISH of human DA neurons. Scale bar, 15 μm. Colors are DAPI (gray), *TH* (green), *CALB1* (yellow), and *ABCB11* (magenta). Outline indicates approximate boundary of DA neuron as identifiable by *TH*. RN, red nucleus; CP, cerebral peduncles; cartesian arrow labels are D, dorsal, V, ventral; M, medial; L, lateral.

out ~500,000 individuals in the UK Biobank, which has an ascertainment bias for healthy individuals, and were absent from our control cohort. This high prevalence in our SCZ cohort of relatively large ~100–500-kb deletions, and the known involvement of germline *NRXN1* mutations in SCZ and other neurodevelopmental disorders, suggests that mosaic deletions of exons 1–5 might also contribute to SCZ risk.

A study characterizing germline *NRXN1* deletions from 19,263 clinical arrays in individuals with neurodevelopmental disease found that most of these events were present in the 5′ end of *NRXN1* and covered exons 1–5.[32] In a case series of germline deletions in *NRXN1* in individuals with more severe developmental disorders,[57] two subjects with severe developmental delay had inherited deletions of exons 1–5. In contrast, germline deletions of *NRXN1* in SCZ are widely distributed throughout the gene[23] rather than being concentrated in the first few exons as in neurodevelopmental disorders.[32,33] This contrast might indicate that germline deletions of exons 1–5 result in more severe developmental phenotypes but, if present in only a fraction of cells, could result in a milder phenotype resembling SCZ.

While the most parsimonious model of pathogenicity of somatic deletions in *NRXN1* exons 1–5 is simple loss of function through deletion of the alpha promoter, the vast diversity of *NRXN1* isoforms warrants further exploration of alternative mechanisms. Our analysis of Hi-C data using hiPSC neurons suggests a potential formation of a cryptic promoter once the *NRXN1* alpha promoter is deleted, potentially forming an N-terminal truncated form of *NRXN1*, leading to a novel dominant-negative mechanism by trapping *NRXN1*α in the cytoplasm. This mechanism is consistent with higher intracellular protein levels of a NRXN1-binding protein CASK in hiPSC lines from SCZ patients with 5′ *NRXN1* deletions.[38] However, further transcriptional and functional experiments could better validate the presence and role of this putative cryptic promoter in *NRXN1* and SCZ biology.

In this study, we also found five early-developmental recurrent somatic deletions in the *ABCB11* transporter gene. These deletions were present only in the SCZ cases diagnosed with TRS, which is defined as nonresponse to at least two anti-psychotic medications[58] and affects ~30% of individuals with SCZ.[59] Genes in this transporter family, including *ABCB11*, have previously been associated with differential response to anti-psychotics.[41] However, the exact mechanism by which mutations in these genes might lead to poor response to anti-psychotics remains unknown.

We show that *ABCB11* is strongly expressed in human DA neurons, specifically within the dorsal stream of the SN. Most anti-psychotic medications used to treat SCZ target DA signaling in the brain, but how DA pathways become abnormal in SCZ remains unclear. Disruption of *ABCB11* could alter the function of this key neuronal circuitry in a relatively cell-type-specific manner. While the exact role of *ABCB11* on DA neuron physiology or excitatory layer 5 neurons is yet unknown, our results suggest this as an area for further inquiry with potential disease relevance.

Limitations of the study

This study has several limitations that represent further areas of research. While we were able to validate most of the variants from DNA we were able to procure, further orthogonal validation is warranted to provide more accurate estimates of the burden of sCNV in SCZ and further characterize sources of potential artifact. The main limitation to validation was obtaining DNA from samples processed at institutions across the world with diverse data-sharing protocols. Our study was also limited to studying variants present in blood at a high cellular fraction, restricting variants characterized to those that might have arisen during early development, which are predicted to also be mosaic in the brain. While this experimental setup provided large sample sizes to test whether SCZ-associated sCNVs were present, future studies using brain-derived tissue might allow further characterization of the potential risk of sCNVs in SCZ. We were also limited in detecting chimeric fusion genes since the SNP density of the array platforms used in this study (~1/3 of SNPs being heterozygous) prevents enough resolution to call these events confidently without a more dedicated method. In addition, the sparsity of clinical and environmental information in our dataset limited our ability to measure interactions of these factors with the burden of sCNV in SCZ, which suggests a potential area of future research. Finally, studying the functional role of the sCNVs in *NRXN1* and *ABCB11*, and somatic variants in general, will require novel mosaic models such as organoids, or animal models, where specific fractions of cells carry the desired events. The development of these models was outside the scope of the current study but presents an exciting future direction. The data presented here represent an initial and preliminary study that is potentially of interest to the field as the role of somatic mutations in general, and sCNV specifically, in disease comes into focus.

CONSORTIA

Brain Somatic Mosaicism Network: Peter J. Park, Daniel Weinberger, John V. Moran, Fred H. Gage, Flora M. Vaccarino, Joseph Gleeson, Gary Mathern, Eric Courchesne, Subhojit Roy, Sara Bizzotto, Michael Coulter, Caroline Dias, Alissa D'Gama, Javier Ganz, Robert Hill, August Yue Huang, Sattar Khoshkhoo, Sonia Kim, Michael Lodato,Michael Miller, Rebeca Borges-Monroy, Rachel Rodin, Zinan Zhou, Craig Bohrson, Chong Chu, Isidro Cortes-Ciriano, Yanmei Dou, Alon Galor, Doga Gulhan, Minseok Kwon, Joe Luquette, Vinay Viswanadham, Attila Jones,Chaggai Rosenbluh, Sean Cho, Ben Langmead, Jeremy Thorpe, Jennifer Erwin, Andrew Jaffe, Michael McConnell,Rujuta Narurkar, Apua Paquola, Jooheon Shin, Richard Straub, Alexej Abyzov, Taejeong Bae, Yeongjun Jang, Yifan Wang, Fred Gage, Sara Linker, Patrick Reed, Meiyan Wang, Alexander Urban, Bo Zhou, Xiaowei Zhu, Reenal Pattni, Aitor Serres Amero, David Juan, Irene Lobon, Tomas Marques-Bonet, Manuel Solis Moruno, Raquel Garcia Perez, Inna Povolotskaya, Eduardo Soriano, Danny Antaki, Dan Averbuj, Laurel Ball, Martin Breuss, Xiaoxu Yang, Changuk Chung, Sarah B. Emery, Diane A. Flasch, Jeffrey M. Kidd, Huira C. Kopera, Kenneth Y. Kwan, Ryan E. Mills, John B. Moldovan, Chen Sun, Xuefang Zhao, Weichen Zhou, Trenton J. Frisbie, Adriana Cherskov, Liana Fasching, Alexandre Jourdon, Sirisha Pochareddy, Soraya Scuderi, Nenad Sestan.

Psychiatric Genomic Consortium: Christian R. Marshall, Daniele Merico, Bhooma Thiruvahindrapuram, Zhouzhi Wang, Stephen W. Scherer, Daniel P Howrigan, Stephan Ripke, Brendan

Bulik-Sullivan, Kai-How Farh, Menachem Fromer, Jacqueline I. Goldstein, Hailiang Huang, Phil Lee, Mark J. Daly, Benjamin M. Neale, Richard A. Belliveau Jr, Sarah E. Bergen, Elizabeth Bevilacqua, Kimberley D. Chambert, Colm O'Dushlaine, Edward M. Scolnick, Jordan W. Smoller, Jennifer L. Moran, Aarno Palotie, Tracey L. Petryshen, Wenting Wu, Douglas S. Greer, Danny Antaki, Aniket Shetty, Madhusudan Gujral, William M. Brandler, Dheeraj Malhotra, Karin V. Fuentes Fajarado, Michelle S. Maile, Peter A. Holmans, Noa Carrera, Nick Craddock, Valentina Escott-Price, Lyudmila Georgieva, Marian L. Hamshere, David Kavanagh, Sophie E. Legge, Andrew J. Pocklington, Alexander L. Richards, Douglas M. Ruderfer, Nigel M. Williams, George Kirov, Michael J. Owen, Dalila Pinto, Guiqing Cai, Kenneth L. Davis, Elodie Drapeau, Joseph I Friedman, Vahram Haroutunian, Elena Parkhomenko, Abraham Reichenberg, Jeremy M. Silverman, Joseph D. Buxbaum, Enrico Domenici, Ingrid Agartz, Srdjan Djurovic, Morten Mattingsdal, Ingrid Melle, Ole A. Andreassen, Erik G. Jönsson, Erik Söderman, Margot Albus, Madeline Alexander, Claudine Laurent, Douglas F. Levinson, Farooq Amin, Joshua Atkins, Murray J. Cairns, Rodney J. Scott, Paul A. Tooney, Jing Qin Wu, Silviu A. Bacanu, Tim B. Bigdeli, Mark A. Reimers, Bradley T. Webb, Aaron R. Wolen, Brandon K. Wormley, Kenneth S. Kendler, Brien P. Riley, Anna K. Kähler, Patrik K. E. Magnusson, Christina M. Hultman, Marcelo Bertalan, Thomas Hansen, Line Olsen, Henrik B. Rasmussen, Thomas Werge, Manuel Mattheisen, Donald W. Black, Richard Bruggeman, Nancy G. Buccola, Randy L. Buckner, Joshua L. Roffman, William Byerley, Wiepke Cahn, René S Kahn, Eric Strengman, Roel A. Ophoff, Vaughan J. Carr, Stanley V. Catts, Frans A. Henskens, Carmel M. Loughland, Patricia T. Michie, Christos Pantelis, Ulrich Schall, Assen V. Jablensky, Brian J. Kelly, Dominique Campion, Rita M. Cantor, Wei Cheng, C. Robert Cloninger, Dragan M Svrakic, David Cohen, Paul Cormican, Gary Donohoe, Derek W. Morris, Aiden Corvin, Michael Gill, Benedicto Crespo-Facorro, James J. Crowley, Martilias S. Farrell, Paola Giusti-Rodríguez, Yunjung Kim, Jin P. Szatkiewicz, Stephanie Williams, David Curtis, Jonathan Pimm, Hugh Gurling, Andrew McQuillin, Michael Davidson, Mark Weiser, Franziska Degenhardt, Andreas J. Forstner, Stefan Herms, Per Hoffmann, Andrea Hofman, Sven Cichon, Markus M. Nöthen, Jurgen Del Favero, Lynn E. DeLisi, Robert W. McCarley, Deborah L. Levy, Raquelle I. Mesholam-Gately, Larry J. Seidman, Dimitris Dikeos, George N. Papadimitriou, Timothy Dinan, Jubao Duan, Alan R. Sanders, Pablo V. Gejman, Elliot S. Gershon, Frank Dudbridge, Peter Eichhammer, Johan Eriksson, Veikko Salomaa, Laurent Essioux, Ayman H. Fanous, James A. Knowles, Michele T. Pato, Carlos N. Pato, Josef Frank, Sandra Meier, Thomas G. Schulze, Jana Strohmaier, Stephanie H. Witt, Marcella Rietschel, Lude Franke, Juha Karjalainen, Robert Freedman, Ann Olincy, Nelson B. Freimer, Shaun M. Purcell, Panos Roussos, Eli A. Stahl, Pamela Sklar, Jordan W. Smoller, Ina Giegling, Annette M. Hartmann, Bettina Konte, Dan Rujescu, Stephanie Godard, Joel N. Hirschhorn, Tune H. Pers, Alkes Price, Tõnu Esko, Jacob Gratten, S. Hong Lee, Peter M. Visscher, Naomi R. Wray, Bryan J. Mowry, Lieuwe de Haan, Carin J. Meijer, Mark Hansen, Masashi Ikeda, Nakao Iwata, Inge Joa, Luba Kalaydjieva, Matthew C. Keller, James L. Kennedy, Clement C. Zai, Jo Knight, Bernard Lerer, Kung-Yee Liang, Jeffrey Lieberman, T. Scott Stroup, Jouko Lönnqvist, Jaana Suvisaari, Brion S. Maher, Wolfgang Maier, Jacques Mallet, Colm McDonald, Andrew M. McIntosh, Douglas H. R. Blackwood, Andres Metspalu, Lili Milani, Vihra Milanova, Younes Mokrab, David A. Collier, Bertram Müller-Myhsok, Kieran C. Murphy, Robin M. Murray, John Powell, Inez Myin-Germeys, Jim Van Os, Igor Nenadic, Deborah A. Nertney, Gerald Nestadt, Ann E. Pulver, Kristin K. Nicodemus, Laura Nisenbaum, Annelie Nordin, Rolf Adolfsson, Eadbhard O'Callaghan, Sang-Yun Oh, F. Anthony O'Neill, Tiina Paunio, Olli Pietiläinen, Diana O. Perkins, Digby Quested, Adam Savitz, Qingqin S. Li, Sibylle G. Schwab, Jianxin Shi, Chris C. A. Spencer, Srinivas Thirumalai, Juha Veijola, John Waddington, Dermot Walsh, Dieter B. Wildenauer, Elvira Bramon, Ariel Darvasi, Danielle Posthuma, David St. Clair, Omar Shanta, Marieke Klein.

STAR★METHODS

Detailed methods are provided in the online version of this paper and include the following:

- KEY RESOURCES TABLE
- RESOURCE AVAILABILITY
  - Lead contact
  - Materials availability
  - Data and code availability
- EXPERIMENTAL MODEL AND SUBJECT DETAILS
  - SNP array data acquisition
  - Data processing
- METHOD DETAILS
  - Variant level quality control
  - Sample-level quality control
  - Event type classification
  - Filtration of mosaic CNV calls
  - Statistical analysis
  - Cell fraction, gene-set, length, and gene number burden analysis
  - Gene set enrichment analysis
  - Permutation test for enrichment of sCNV overlapping exons 1–5 of NRXN1
  - Germline CNV analyses
  - Breakpoint microhomology analysis
  - *In situ* Hi-C from hiPSC-derived neurons
  - Hi-C read alignment
  - Preprocessing for variant calling
  - Variant calling for Hi-C analysis
  - Haplotype phasing for Hi-C analysis
  - Hi-C matrix construction and visualization
  - Analysis of human postmortem snRNA-seq datasets
  - Single-molecule *in situ* hybridization (smFISH) and imaging of postmortem human nigra

SUPPLEMENTAL INFORMATION

Supplemental information can be found online at https://doi.org/10.1016/j.xgen.2023.100356.

ACKNOWLEDGMENTS

E.A.M. is supported by the Harvard/MIT MD-PhD program (T32GM007753), the Biomedical Informatics and Data Science Training Program

(T15LM007092), and the Ruth L. Kirschstein NRSA F31 Fellowship (F31MH124292). G.G. is supported by NIH grant R01HG006855, NIH grant R01MH104964, and the Stanley Center for Psychiatric Research. S.A. was supported by NIH grant R01MH106056 and S.A., A.C., and C.A.W. were supported by the NIMH grant (U01MH106883) through the Brain Somatic Mosaicism Network (BSMN). C.A.W. is an investigator of the Howard Hughes Medical Institute. C.A.W. and E.A.L. are supported by the Allen Frontiers Program through the Allen Discovery Center for Human Brain Evolution. E.A.L. is supported by NIH grants (K01 AG051791, DP2 AG072437, and R01AG070921) and the SUHF foundation. J.S. is supported by NIH grants (MH113715, MH119746, MH109501, and MH119746). J.E.P.-C. and K.J.B. are supported by a Chan Zuckerberg Initiative grant (2020-221479). J.E.P.-C. is supported by NIH grants (DP1OD031253, R01NS-114226, R01MH12026, and U01DK127405). P.-R.L. is supported by NIH grant DP2 ES030554 and a Burroughs Wellcome Fund Career Award at the Scientific Interfaces. T.K. is supported by F30AG069446-01. E.Z.M. is supported by DP2AG058488, U01MH124602, and Chan Zuckerberg Initiative (no. 2017-175259).

AUTHOR CONTRIBUTIONS

E.A.M. and C.A.W. conceived and designed the study. E.A.M. designed and implemented the statistical methods. E.A.M. performed computational analyses, with assistance from M.A.S. and G.G. J.S. curated the data and facilitated access. S.M. and A.C. facilitated acquisition of samples for whole-genome sequencing validation. J.T.R.W., M.O., and P.S. facilitated clinical and genomic data procurement for validation and interpretation. P.R., S.A., and K.J.B. generated the Hi-C data. T.G.G. and J.E.P.-C. analyzed and interpreted the Hi-C data. E.F. and K.J.B. generated and characterized the hiPSCs/neurons. T.K., S.B., and E.Z.M. contributed and analyzed the *ABCB11* snRNA-seq data. E.A.L., P.-R.L., S.A.M., and J.S. provided comments and guidance throughout. E.A.M., E.A.L., and C.A.W. wrote the manuscript.

DECLARATION OF INTERESTS

The authors declare no competing interests.

INCLUSION AND DIVERSITY

We support inclusive, diverse, and equitable conduct of research.

Received: November 30, 2021
Revised: February 21, 2022
Accepted: June 9, 2023
Published: July 6, 2023

REFERENCES

1. Kirov, G., Pocklington, A.J., Holmans, P., Ivanov, D., Ikeda, M., Ruderfer, D., Moran, J., Chambert, K., Toncheva, D., Georgieva, L., et al. (2012). De novo CNV analysis implicates specific abnormalities of postsynaptic signalling complexes in the pathogenesis of schizophrenia. Mol. Psychiatry *17*, 142–153. https://doi.org/10.1038/mp.2011.154.
2. Kirov, G., Rujescu, D., Ingason, A., Collier, D.A., O'Donovan, M.C., and Owen, M.J. (2009). Neurexin 1 (NRXN1) Deletions in Schizophrenia. Schizophr. Bull. *35*, 851–854. https://doi.org/10.1093/schbul/sbp079.
3. Sherman, M.A., Rodin, R.E., Genovese, G., Dias, C., Barton, A.R., Mukamel, R.E., Berger, B., Park, P.J., Walsh, C.A., and Loh, P.R. (2021). Large mosaic copy number variations confer autism risk. Nat. Neurosci. *24*, 197–203. https://doi.org/10.1038/s41593-020-00766-5.
4. Maury, E.A., and Walsh, C.A. (2021). Somatic copy number variants in neuropsychiatric disorders. Curr. Opin. Genet. Dev. *68*, 9–17. https://doi.org/10.1016/j.gde.2020.12.013.
5. Ruderfer, D.M., Chambert, K., Moran, J., Talkowski, M., Chen, E.S., Gigek, C., Gusella, J.F., Blackwood, D.H., Corvin, A., Gurling, H.M., et al. (2013). Mosaic copy number variation in schizophrenia. Eur. J. Hum. Genet. *21*, 1007–1011. https://doi.org/10.1038/ejhg.2012.287.
6. King, D.A., Jones, W.D., Crow, Y.J., Dominiczak, A.F., Foster, N.A., Gaunt, T.R., Harris, J., Hellens, S.W., Homfray, T., Innes, J., et al. (2015). Mosaic structural variation in children with developmental disorders. Hum. Mol. Genet. *24*, 2733–2745. https://doi.org/10.1093/hmg/ddv033.
7. Bae, T., Fasching, L., Wang, Y., Shin, J.H., Suvakov, M., Jang, Y., Norton, S., Dias, C., Mariani, J., Jourdon, A., et al. (2022). Analysis of somatic mutations in 131 human brains reveals aging-associated hypermutability. Science *377*, 511–517. https://doi.org/10.1126/SCIENCE.ABM6222/SUPPL_FILE/SCIENCE.ABM6222_MDAR_REPRODUCIBILITY_CHECKLIST.PDF.
8. Bae, T., Wang, Y., Vaccarino, F.M., and Abyzov, A. (2022). Somatic genomic mosaicism in the brain during aging: Scratching the surface. Clin. Transl. Med. *12*, e1138. https://doi.org/10.1002/CTM2.1138.
9. Poduri, A., Evrony, G.D., Cai, X., Elhosary, P.C., Beroukhim, R., Lehtinen, M.K., Hills, L.B., Heinzen, E.L., Hill, A., Hill, R.S., et al. (2012). Somatic Activation of AKT3 Causes Hemispheric Developmental Brain Malformations. Neuron *74*, 41–48. https://doi.org/10.1016/j.neuron.2012.03.010.
10. Conti, V., Pantaleo, M., Barba, C., Baroni, G., Mei, D., Buccoliero, A.M., Giglio, S., Giordano, F., Baek, S.T., Gleeson, J.G., and Guerrini, R. (2015). Focal dysplasia of the cerebral cortex and infantile spasms associated with somatic 1q21.1-q44 duplication including the *AKT3* gene. Clin. Genet. *88*, 241–247. https://doi.org/10.1111/cge.12476.
11. López-Rivera, J.A., Leu, C., Macnee, M., Khoury, J., Hoffmann, L., Coras, R., Kobow, K., Bhattarai, N., Pérez-Palma, E., Hamer, H., et al. (2022). The genomic landscape across 474 surgically accessible epileptogenic human brain lesions. Brain. https://doi.org/10.1093/BRAIN/AWAC376.
12. Kushima, I., Aleksic, B., Nakatochi, M., Mori, D., Iwata, N., and Ozaki, N. (2018). Comparative Analyses of Copy-Number Variation in Autism Spectrum Disorder and Schizophrenia Reveal Etiological Overlap and Biological Insights. Cell Rep. *24*, 2838–2856, Etiological overlap Orange: Intellectual disability + Patients without pathogenic CNVs. https://doi.org/10.1016/j.celrep.2018.08.022.
13. Lodato, M.A., Woodworth, M.B., Lee, S., Evrony, G.D., Mehta, B.K., Karger, A., Lee, S., Chittenden, T.W., D'Gama, A.M., Cai, X., et al. (2015). Somatic mutation in single human neurons tracks developmental and transcriptional history. Science *350*, 94–98. https://doi.org/10.1126/science.aab1785.
14. Bizzotto, S., Dou, Y., Ganz, J., Doan, R.N., Kwon, M., Bohrson, C.L., Kim, S.N., Bae, T., Abyzov, A., et al.; NIMH Brain Somatic Mosaicism Network (2021). Landmarks of human embryonic development inscribed in somatic mutations. Science *371*, 1249–1253. https://doi.org/10.1126/SCIENCE.ABE1544/SUPPL_FILE/ABE1544-BIZZOTTO-SM.PDF.
15. Bae, T., Tomasini, L., Mariani, J., Zhou, B., Roychowdhury, T., Franjic, D., Pletikos, M., Pattni, R., Chen, B.-J., Venturini, E., et al. (2018). Different mutational rates and mechanisms in human cells at pregastrulation and neurogenesis. Science *359*, 550–555. https://doi.org/10.1126/SCIENCE.AAN8690.
16. Fasching, L., Jang, Y., Tomasi, S., Schreiner, J., Tomasini, L., Brady, M.V., Bae, T., Sarangi, V., Vasmatzis, N., Wang, Y., et al. (2021). Early developmental asymmetries in cell lineage trees in living individuals. Science *371*, 1245–1248. https://doi.org/10.1126/SCIENCE.ABE0981/SUPPL_FILE/ABE0981_TABLE_S3.XLSX.
17. Ju, Y.S., Martincorena, I., Gerstung, M., Petljak, M., Alexandrov, L.B., Rahbari, R., Wedge, D.C., Davies, H.R., Ramakrishna, M., Fullam, A., et al. (2017). Somatic mutations reveal asymmetric cellular dynamics in the early human embryo. Nature *543*, 714–718. https://doi.org/10.1038/nature21703.
18. Loh, P.-R., Genovese, G., Handsaker, R.E., Finucane, H.K., Reshef, Y.A., Palamara, P.F., Birmann, B.M., Talkowski, M.E., Bakhoum, S.F., McCarroll, S.A., and Price, A.L. (2018). Insights into clonal haematopoiesis from 8,342 mosaic chromosomal alterations. Nature *559*, 350–355. https://doi.org/10.1038/s41586-018-0321-x.

19. Loh, P.R., Genovese, G., and McCarroll, S.A. (2020). Monogenic and polygenic inheritance become instruments for clonal selection. Nature *584*, 136–141. https://doi.org/10.1038/s41586-020-2430-6.

20. Zekavat, S.M., Lin, S.H., Bick, A.G., Liu, A., Paruchuri, K., Wang, C., Uddin, M.M., Ye, Y., Yu, Z., Liu, X., et al. (2021). Hematopoietic mosaic chromosomal alterations increase the risk for diverse types of infection. Nat. Med. *27*, 1012–1024. https://doi.org/10.1038/S41591-021-01371-0.

21. Terao, C., Suzuki, A., Momozawa, Y., Akiyama, M., Ishigaki, K., Yamamoto, K., Matsuda, K., Murakami, Y., McCarroll, S.A., Kubo, M., et al. (2020). Chromosomal alterations among age-related haematopoietic clones in Japan. Nature *584*, 130–135. https://doi.org/10.1038/s41586-020-2426-2.

22. Saiki, R., Momozawa, Y., Nannya, Y., Nakagawa, M.M., Ochi, Y., Yoshizato, T., Terao, C., Kuroda, Y., Shiraishi, Y., Chiba, K., et al. (2021). Combined landscape of single-nucleotide variants and copy number alterations in clonal hematopoiesis. Nat. Med. *27*, 1239–1249. https://doi.org/10.1038/S41591-021-01411-9.

23. Marshall, C.R., Howrigan, D.P., Merico, D., Thiruvahindrapuram, B., Wu, W., Greer, D.S., Antaki, D., Shetty, A., Holmans, P.A., Pinto, D., et al. (2017). Contribution of copy number variants to schizophrenia from a genome-wide study of 41,321 subjects. Nat. Genet. *49*, 27–35. https://doi.org/10.1038/ng.3725.

24. Qin, N., Wang, C., Chen, C., Yang, L., Liu, S., Xiang, J., Xie, Y., Liang, S., Zhou, J., Xu, X., et al. (2022). Association of the interaction between mosaic chromosomal alterations and polygenic risk score with the risk of lung cancer: an array-based case-control association and prospective cohort study. Lancet Oncol. *23*, 1465–1474. https://doi.org/10.1016/S1470-2045(22)00600-3.

25. Brown, D.W., Lin, S.H., Loh, P.R., Chanock, S.J., Savage, S.A., and Machiela, M.J. (2020). Genetically predicted telomere length is associated with clonal somatic copy number alterations in peripheral leukocytes. PLoS Genet. *16*, e1009078. https://doi.org/10.1371/JOURNAL.PGEN.1009078.

26. Lek, M., Karczewski, K.J., Minikel, E.V., Samocha, K.E., Banks, E., Fennell, T., O'Donnell-Luria, A.H., Ware, J.S., Hill, A.J., Cummings, B.B., et al. (2016). Analysis of protein-coding genetic variation in 60,706 humans. Nature *536*, 285–291. https://doi.org/10.1038/nature19057.

27. Singh, T., Poterba, T., Curtis, D., Akil, H., Al Eissa, M., Barchas, J.D., Bass, N., Bigdeli, T.B., Breen, G., Bromet, E.J., et al. (2022). Rare coding variants in ten genes confer substantial risk for schizophrenia. Nat *604*, 509–516. https://doi.org/10.1038/s41586-022-04556-w.

28. Weiss, L.A., Shen, Y., Korn, J.M., Arking, D.E., Miller, D.T., Fossdal, R., Saemundsen, E., Stefansson, H., Ferreira, M.A.R., Green, T., et al. (2008). Association between Microdeletion and Microduplication at 16p11.2 and Autism. N. Engl. J. Med. *358*, 667–675.

29. Arinami, T. (2006). Analyses of the associations between the genes of 22q11 deletion syndrome and schizophrenia. J. Hum. Genet. *51*, 1037–1045. https://doi.org/10.1007/s10038-006-0058-5.

30. Gothelf, D., Law, A.J., Frisch, A., Chen, J., Zarchi, O., Michaelovsky, E., Ren-Patterson, R., Lipska, B.K., Carmel, M., Kolachana, B., et al. (2014). Biological effects of COMT haplotypes and psychosis risk in 22q11.2 deletion syndrome. Biol. Psychiatry *75*, 406–413. https://doi.org/10.1016/j.biopsych.2013.07.021.

31. Flaherty, E., Zhu, S., Barretto, N., Cheng, E., Deans, P.J.M., Fernando, M.B., Schrode, N., Francoeur, N., Antoine, A., Alganem, K., et al. (2019). Neuronal impact of patient-specific aberrant NRXN1α splicing. Nat. Genet. *51*, 1679–1690. https://doi.org/10.1038/s41588-019-0539-z.

32. Lowther, C., Speevak, M., Armour, C.M., Goh, E.S., Graham, G.E., Li, C., Zeesman, S., Nowaczyk, M.J.M., Schultz, L.A., Morra, A., et al. (2017). Molecular characterization of NRXN1 deletions from 19,263 clinical microarray cases identifies exons important for neurodevelopmental disease expression. Genet. Med. *19*, 53–61. https://doi.org/10.1038/gim.2016.54.

33. Cosemans, N., Vandenhove, L., Vogels, A., Devriendt, K., Van Esch, H., Van Buggenhout, G., Olivié, H., De Ravel, T., Ortibus, E., Legius, E., et al. (2020). The clinical relevance of intragenic NRXN1 deletions. J. Med. Genet. *57*, 347–355. https://doi.org/10.1136/jmedgenet-2019-106448.

34. Roadmap Epigenomics Consortium; Kundaje, A., Meuleman, W., Ernst, J., Bilenky, M., Yen, A., Heravi-Moussavi, A., Kheradpour, P., Zhang, Z., Wang, J., et al. (2015). Integrative analysis of 111 reference human epigenomes. Nature *518*, 317–330. https://doi.org/10.1038/nature14248.

35. Yang, L., Luquette, L.J., Gehlenborg, N., Xi, R., Haseley, P.S., Hsieh, C.H., Zhang, C., Ren, X., Protopopov, A., Chin, L., et al. (2013). Diverse mechanisms of somatic structural variations in human cancer genomes. Cell *153*, 919–929. https://doi.org/10.1016/j.cell.2013.04.010.

36. Kidd, J.M., Graves, T., Newman, T.L., Fulton, R., Hayden, H.S., Malig, M., Kallicki, J., Kaul, R., Wilson, R.K., and Eichler, E.E. (2010). A Human Genome Structural Variation Sequencing Resource Reveals Insights into Mutational Mechanisms. Cell *143*, 837–847. https://doi.org/10.1016/j.cell.2010.10.027.

37. Zhang, F., Khajavi, M., Connolly, A.M., Towne, C.F., Batish, S.D., and Lupski, J.R. (2009). The DNA replication FoSTeS/MMBIR mechanism can generate genomic, genic and exonic complex rearrangements in humans. Nat. Genet. *41*, 849–853. https://doi.org/10.1038/ng.399.

38. Pak, C., Danko, T., Mirabella, V.R., Wang, J., Liu, Y., Vangipuram, M., Grieder, S., Zhang, X., Ward, T., Huang, Y.-W.A., et al. (2021). Cross-platform validation of neurotransmitter release impairments in schizophrenia patient-derived NRXN1-mutant neurons. Proc. Natl. Acad. Sci. USA *118*, 2025598118. https://doi.org/10.1073/PNAS.2025598118.

39. Bompadre, O., and Andrey, G. (2019). Chromatin topology in development and disease. Curr. Opin. Genet. Dev. *55*, 32–38. https://doi.org/10.1016/J.GDE.2019.04.007.

40. Halvorsen, M., Huh, R., Oskolkov, N., Wen, J., Netotea, S., Giusti-Rodriguez, P., Karlsson, R., Bryois, J., Nystedt, B., Ameur, A., et al. (2020). Increased burden of ultra-rare structural variants localizing to boundaries of topologically associated domains in schizophrenia. Nat. Commun. *11*, 1842. https://doi.org/10.1038/s41467-020-15707-w.

41. Vita, A., Minelli, A., Barlati, S., Deste, G., Giacopuzzi, E., Valsecchi, P., Turrina, C., and Gennarelli, M. (2019). Treatment-resistant schizophrenia: Genetic and neuroimaging correlates. Front. Pharmacol. *10*, 402. https://doi.org/10.3389/fphar.2019.00402.

42. Gonzalez-Covarrubias, V., Martínez-Magaña, J.J., Coronado-Sosa, R., Villegas-Torres, B., Genis-Mendoza, A.D., Canales-Herrerias, P., Nicolini, H., and Soberón, X. (2016). Exploring Variation in Known Pharmacogenetic Variants and its Association with Drug Response in Different Mexican Populations. Pharm. Res. (N. Y.) *33*, 2644–2652. https://doi.org/10.1007/s11095-016-1990-5.

43. Strautnieks, S.S., Bull, L.N., Knisely, A.S., Kocoshis, S.A., Dahl, N., Arnell, H., Sokal, E., Dahan, K., Childs, S., Ling, V., et al. (1998). A gene encoding a liver-specific ABC transporter is mutated in progressive familial intrahepatic cholestasis. Nat. Genet. *20*, 233–238. https://doi.org/10.1038/3034.

44. Knisely, A.S., Strautnieks, S.S., Meier, Y., Stieger, B., Byrne, J.A., Portmann, B.C., Bull, L.N., Pawlikowska, L., Bilezikçi, B., Ozçay, F., et al. (2006). Hepatocellular carcinoma in ten children under five years of age with bile salt export pump deficiency. Hepatology *44*, 478–486. https://doi.org/10.1002/HEP.21287.

45. Van Mil, S.W.C., Van Der Woerd, W.L., Van Der Brugge, G., Sturm, E., Jansen, P.L.M., Bull, L.N., Van Den Berg, I.E.T., Berger, R., Houwen, R.H.J., and Klomp, L.W.J. (2004). Benign recurrent intrahepatic cholestasis type 2 is caused by mutations in ABCB11. Gastroenterology *127*, 379–384. https://doi.org/10.1053/J.GASTRO.2004.04.065.

46. Jansen, P.L., Strautnieks, S.S., Jacquemin, E., Hadchouel, M., Sokal, E.M., Hooiveld, G.J., Koning, J.H., De Jager-Krikken, A., Kuipers, F., Stellaard, F., et al. (1999). Hepatocanalicular bile salt export pump deficiency in patients with progressive familial intrahepatic cholestasis. Gastroenterology *117*, 1370–1379. https://doi.org/10.1016/S0016-5085(99)70287-8.

47. Ortiz, D.F., Moseley, J., Calderon, G., Swift, A.L., Li, S., and Arias, I.M. (2004). Identification of HAX-1 as a protein that binds bile salt export

CellPress
OPEN ACCESS

protein and regulates its abundance in the apical membrane of Madin-Darby canine kidney cells. J. Biol. Chem. *279*, 32761–32770. https://doi.org/10.1074/jbc.M404337200.

48. Alogaili, F., Chinnarasu, S., Jaeschke, A., Kranias, E.G., Hui, D.Y., and Pessin, J.E. (2020). Hepatic HAX-1 inactivation prevents metabolic diseases by enhancing mitochondrial activity and bile salt export. J. Biol. Chem. *295*, 4631–4646. https://doi.org/10.1074/jbc.RA119.012361.
49. Schizophrenia Working Group of the Psychiatric Genomics Consortium; Ripke, S., Neale, B.M., Corvin, A., Walters, J.T.R., Farh, K.-H., Holmans, P.A., Lee, P., Bulik-Sullivan, B., Collier, D.A., et al. (2014). Biological insights from 108 schizophrenia-associated genetic loci. Nature *511*, 421–427. https://doi.org/10.1038/nature13595.
50. Hamshere, M.L., Walters, J.T.R., Smith, R., Richards, A.L., Green, E., Grozeva, D., Jones, I., Forty, L., Jones, L., Gordon-Smith, K., et al. (2013). Genome-wide significant associations in schizophrenia to ITIH3/4, CACNA1C and SDCCAG8, and extensive replication of associations reported by the Schizophrenia PGC. Mol. Psychiatry *18*, 708–712. https://doi.org/10.1038/mp.2012.67.
51. Song, X., Beck, C.R., Du, R., Campbell, I.M., Coban-Akdemir, Z., Gu, S., Breman, A.M., Stankiewicz, P., Ira, G., Shaw, C.A., and Lupski, J.R. (2018). Predicting human genes susceptible to genomic instability associated with Alu/Alu-mediated rearrangements. Genome Res. *28*, 1228–1242. https://doi.org/10.1101/GR.229401.117.
52. Kamath, T., Abdulraouf, A., Burris, S.J., Langlieb, J., Gazestani, V., Nadaf, N.M., Balderrama, K., Vanderburg, C., and Macosko, E.Z. (2022). Single-cell genomic profiling of human dopamine neurons identifies a population that selectively degenerates in Parkinson's disease. Nat. Neurosci. *25*, 588–595. https://doi.org/10.1038/s41593-022-01061-1.
53. Bakken, T.E., Jorstad, N.L., Hu, Q., Lake, B.B., Tian, W., Kalmbach, B.E., Crow, M., Hodge, R.D., Krienen, F.M., Sorensen, S.A., et al. (2021). Comparative cellular analysis of motor cortex in human, marmoset and mouse. Nat *598*, 111–119. https://doi.org/10.1038/s41586-021-03465-8.
54. Haber, S.N. (2014). The place of dopamine in the cortico-basal ganglia circuit. Neuroscience *282*, 248–257. https://doi.org/10.1016/J.NEUROSCIENCE.2014.10.008.
55. Zhang, Y., Larcher, K.M.H., Misic, B., and Dagher, A. (2017). Anatomical and functional organization of the human substantia Nigra and its connections. Elife *6*, e26653. https://doi.org/10.7554/ELIFE.26653.
56. Perez-Costas, E., Melendez-Ferro, M., and Roberts, R.C. (2010). Basal ganglia pathology in schizophrenia: dopamine connections and anomalies. J. Neurochem. *113*, 287–302. https://doi.org/10.1111/J.1471-4159.2010.06604.X.
57. Ching, M.S.L., Shen, Y., Tan, W.H., Jeste, S.S., Morrow, E.M., Chen, X., Mukaddes, N.M., Yoo, S.Y., Hanson, E., Hundley, R., et al. (2010). Deletions of NRXN1 (neurexin-1) predispose to a wide spectrum of developmental disorders. Am. J. Med. Genet. B Neuropsychiatr. Genet. *153B*, 937–947. https://doi.org/10.1002/ajmg.b.31063.
58. National Institute of Health and Clinical Excellence (2014). Psychosis and Schizophrenia in Adults (NICE Guidel. treament Manag.), pp. 74–80.
59. Meltzer, H.Y. (1997). Treatment-resistant schizophrenia - The role of clozapine. Curr. Med. Res. Opin. *14*, 1–20. https://doi.org/10.1185/03007999709113338.
60. Gel, B., Díez-Villanueva, A., Serra, E., Buschbeck, M., Peinado, M.A., and Malinverni, R. (2016). regioneR: an R/Bioconductor package for the association analysis of genomic regions based on permutation tests. Bioinformatics *32*, 289–291. https://doi.org/10.1093/BIOINFORMATICS/BTV562.
61. Thorvaldsdóttir, H., Robinson, J.T., and Mesirov, J.P. (2013). Integrative Genomics Viewer (IGV): High-performance genomics data visualization and exploration. Brief. Bioinform. *14*, 178–192. https://doi.org/10.1093/bib/bbs017.
62. Bates, D.W., Mächler, M., Bolker, B.M., and Walker, S.C. (2015). Fitting linear mixed-effects models using lme4. BMJ Qual. Saf. *24*, 1–3. https://doi.org/10.18637/jss.v067.i01.
63. Kuznetsova, A., Brockhoff, P.B., and Christensen, R.H.B. (2017). lmerTest Package: Tests in Linear Mixed Effects Models. J. Stat. Softw. *82*, 1–26. https://doi.org/10.18637/jss.v082.i13.
64. Loh, P.R., Danecek, P., Palamara, P.F., Fuchsberger, C., A Reshef, Y., K Finucane, H., Schoenherr, S., Forer, L., McCarthy, S., Abecasis, G.R., et al. (2016). Reference-based phasing using the Haplotype Reference Consortium panel. Nat. Genet. *48*, 1443–1448. https://doi.org/10.1038/ng.3679.
65. Vattathil, S., and Scheet, P. (2013). Haplotype-based profiling of subtle allelic imbalance with SNP arrays. Genome Res. *23*, 152–158. https://doi.org/10.1101/gr.141374.112.
66. Raychaudhuri, S., Korn, J.M., McCarroll, S.A., International Schizophrenia Consortium; Altshuler, D., Sklar, P., Purcell, S., and Daly, M.J. (2010). Accurately Assessing the Risk of Schizophrenia Conferred by Rare Copy-Number Variation Affecting Genes with Brain Function. PLoS Genet. *6*, e1001097. https://doi.org/10.1371/journal.pgen.1001097.

STAR★METHODS

KEY RESOURCES TABLE

REAGENT or RESOURCE	SOURCE	IDENTIFIER
**Critical commercial assays**		
Hi-C Kit	Arima	N/A
SuperFrost Plus slides		N/A
Probe hybridization buffer	Molecular Instruments	N/A
Probe amplification buffer	Molecular Instruments	N/A
5xSSCT (20% Tween)	ThermoFisher Scientific	Catalog # 15557044
Hairpins	Molecular Instruments	N/A
Probes	Molecular Instrument	Costume made based on accession number see STAR Methods
**Biological sample data**		
healthy adult postmortem midbrain block	Sepulveda Human Brain and Spinal Fluid Resource Center	http://brainbank.ucla.edu/
**Deposited data**		
Individual level SNP-array data	Psychiatric Genomic Consortium	https://www.med.unc.edu/pgc/shared-methods/how-to/
Filtered sCNV callset	This paper	data listed in Filtered sCNV callset is in Table S2
Top 20% brain expressed genes	GTEx	https://www.gtexportal.org/home/datasets
Synaptic genes	SynaptomeDB	http://metamoodics.org/SynaptomeDB/index.php
gnomAD constrain statistics	gnomAD	https://gnomad.broadinstitute.org/downloads
HapMap variants v3.3		https://www.sanger.ac.uk/resources/downloads/human/hapmap3.html
1000 Genomes "Omni" platform variants v2.5		https://www.internationalgenome.org/category/omni/
Whole Genome Sequencing data	Broad Institute	Sequencing data will be uploaded to the NIMH Data Archive after publication.
**Experimental models: Cell lines**		
hiPSC cell lines:<br>Control NSB2607-2 (2607 clone 1)<br>5′ deletion NSB973-5 (973 clone 1)	Flaherty et al.[31]	N/A
**Software and algorithms**		
MoChA	Loh et al.,[18] Loh et al.[19]	https://github.com/freeseek/mocha
R v 4.0.3	R Core Team	https://www.r-project.org
regioneR (R package)	Gel et al.[60]	https://bioconductor.org/packages/release/bioc/html/regioneR.html
IGV	Thorvaldsdottir et al.[61]	https://software.broadinstitute.org/software/igv/download
Lme4 (R package)	Bates et al.[62]	https://cran.r-project.org/web/packages/lme4/index.html
lmerTest (R package)	Kuznetsova et al.[63]	https://cran.r-project.org/web/packages/lmerTest/index.html
Python v3.6.12	Python Core Team	https://www.python.org/
BWA mem v0.7.17-r1188		https://github.com/lh3/bwa
GATK		https://gatk.broadinstitute.org/hc/en-us

(*Continued on next page*)

Continued

REAGENT or RESOURCE	SOURCE	IDENTIFIER
HapCUT2		https://github.com/vibansal/HapCUT2
PyMOL		https://pymol.org/2/
Other		
Code for main figures and analysis	This paper; Zenodo	emauryg/SCZ_sCNV_paper_repo: Publication release (v1.0.0). Zenodo. https://doi.org/10.5281/zenodo.7778664
PyMOL was used for ABCB11 schematic in Figure 4 using PBID: 6LR0		N/A

RESOURCE AVAILABILITY

Lead contact

Further information requests for resources and reagents should be directed to and will be fulfilled by lead contact, Christopher A. Walsh (christopher.walsh@childrens.harvard.edu).

Materials availability

All unique/stable reagents generated in this study are available from the lead contact with a completed materials transfer agreement.

Data and code availability

- Individual level SNP-array data is part of the Psychiatric Genomic Consortium with the corresponding privacy agreement. Access can be provided by applying through this website (https://www.med.unc.edu/pgc/shared-methods/how-to/). Whole genome sequncing data for validation experiments will be uploaded to the NIMH Data Archive after publication NDA: (https://nda.nih.gov/).
- Filtered sCNV callset is inTable S2.
- Scripts used to generate the main figures and analyses are available in a frozen Zenodo repository Zenodo: https://doi.org/10.5281/zenodo.7778664.
- PyMOL was used for ABCB11 schematic in Figure 4 using PBID: 6LR0.
- Any additional information required to reanalyze the data reported in this paper is available.

EXPERIMENTAL MODEL AND SUBJECT DETAILS

SNP array data acquisition

Allelic intensity data for cases and controls were obtained from the Psychiatric Genomic Consortium (PGC) CNV working group. The exact details of the data generation were previously described,[23] removing samples derived from cell lines. SNP array data consisting of 13,464 SCZ cases and 12,722 controls was obtained. These data were profiled with the Illumina OmniExpress, OmniExpress plus exome chip, Illum610K, and Affymetrix SNP6.0 arrays. For each determined position the B allele frequency (BAF; proportion of B allele), Log-R ratio (LRR; total genotyping intensity of A and B alleles), and genotype calls, were calculated.

Data processing

The genotypes from the SNPs from the arrays were phased using the Eagle2[64] software. Then, the BCFtools plug-in MoChA (2021-01-20 release) was used to confidently call mosaic CNVs, by taking advantage of long-range haplotype phasing of heterozygous SNP sites and BAF estimates of genotype array data. Genotyping and intensity data from Illumina platforms were distributed by the PGC in the Illumina GenomeStudio Final Report format, with the genomic positions genotyped using the hg18 human reference genome. To convert the Final Report format to VCF format, the rsID numbers were used to liftover coordinates to hg19, discarding positions without rsID, similar to Sherman et al.[3] Custom scripts were used to transform Final Reports to binary VCF format, and Illumina's TOP-BOT format was converted to dbSNP REF-ALT format using a modified version of BCFtools plug-in fix-ref. MoChA calculates cell fraction from BAF as follows:

$$|0.5 - 1/CN| = \Delta BAF;\ CF = |CN - 2|$$

where CN is the copy number and ΔBAF is the deviation of B allele fraction compared to 0.50. This equation is valid for gains and losses.

METHOD DETAILS

Variant level quality control

In accordance with the suggestions of the MoChA processing pipeline, the following variants were filtered out: more than 2% genotypes missing, evidence of excess heterozygosity (p < 1e-6, Hardy-Weinberg equilibrium test), correlation of autosomal genotypes with sex (Fisher exact test comparing number of 0/0 genotypes vs. number of 1/1 genotypes in males and females), variants falling within segmental duplications with low divergence (<2%). This variant-level QC was performed on each separate batch.

Sample-level quality control

In order to filter out samples with contamination from another individual two statistics were calculated: BAF concordance and BAF autocorrelation. Briefly, BAF concordance calculates the probability that an adjacent heterozygous SNP has a deviation from a BAF of 0.5 given that the previous heterozygous site had the same deviation from 0.5.[65] BAF autocorrelation is the correlation of the BAF statistic at consecutive heterozygous sites once adjusted for the genotype phase. Samples with contamination with DNA from another individual would be expected to have a BAF concordance >0.5 and BAF autocorrelation >0 because of allelic intensities correlated at variants within haplotypes shared between sample DNA and contaminated DNA. Samples with BAF concordance >0.51 or BAF autocorrelation >0.03 were removed.

Event type classification

An Expectation Maximization algorithm was applied to classify events as either a Gain, Loss, or CN-LOH. The algorithm determines the slopes that characterizes the relationship between the deviation of the LRR from 0 $|\Delta \mathrm{LRR}|$, and the BAF deviation from 0.5, $|\Delta BAF|$. In other words, the events are classified based on the optimization of linear regression parameters described by $|\Delta LRR| = |\Delta BAF|\beta_c + \epsilon$, where $c \in \{Gain, Loss, CN - LOH\}$, β_c is the slope for each event type, $\epsilon \sim \mathrm{N}(0, \sigma_c^2)$ is the error for each event-type clustering.

To further enhance the robustness of the classification method, we used the fact that CN-LOH events are expected to be less common within the chromosomes compared to events that extend to the telomeres. Since CN-LOH events are thought to arise during mitotic recombination, for them to occur within a chromosome would require a double crossover, which is highly unlikely. To incorporate this information into the classification model, we estimated the frequency using the UK Biobank sCNV calls[18,19] for of each event type occurring on telomeres and interstitially. These frequencies were used as priors to multiply the likelihoods for each event type, resulting in posterior probabilities. The computation for each event S_i is as follows: Let $X = |\Delta BAF|$ and $Y = |\Delta LRR|$, then $\Pr(S_i = c \mid L_i, X_i, Y_i) \propto \Pr(L_i)\, e^{-(Y_i - X_i \beta_c)^2 / 2\sigma_c^2}$, where L_i is an indicator of whether the event involves a telomere, and c is defined as above. This estimation is calculated for each event type and then normalized to sum to one.

Filtration of mosaic CNV calls

Filtration was focused on removing potential germline events and events likely to arise due to age-related clonal hematopoiesis, as well as artifacts. We required events to have a log10-odds >10 for the model based on BAF and phase, which measures how much more likely the data for a given segment of DNA is consistent with a non-diploid model than a diploid model. Events that were classified as copy number polymorphism (known CNV polymorphisms in 1000 Genomes Project) by MoChA were filtered out as possible germline events. We further excluded events that had a reciprocal overlap with events from control samples or with any CNVs reported in the 1000 Genomes project by >50%. Events that overlapped >50% with germline events previously identified in the same sample by the PGC[23] were also removed for duplications, since small duplications with high BAF deviations can be mistakenly identified as somatic variants. Copy number state was taken into consideration when calculating overlaps, i.e. overlap between gains and losses were not considered. Calls with an estimated cell fraction of 1 were also removed. For gains, we further removed any events with a deviation in BAF greater than 0.10 to have a conservative assurance that germline gains were not misclassified as mosaic, as germline gains tend to be small and produce large deviations from the a BAF of about 1/6.[18]

Finally, since most of our datasets did not include age information for individuals besides the broad estimate of being younger than 40, we used a conservative approach to remove events that could have risen from clonal hematopoiesis. CN-LOH events were fully excluded from any downstream analysis as these events have been shown to be largely enriched in clonal hematopoiesis events.[18] We also removed sCNVs that contained loci commonly altered within the immune system, specifically IGH (chr14:105,000,000–108,000,000) and IGL (chr22:22,000,000–40,000,000). We also excluded CNVs within the extended MHC region (chr6:19,000,000–40,000,000). In addition, we removed deletion involving the following loci that are frequently affected by clonal hematopoiesis: 20q11, *DNMT3A*, *TET2*, 13q14, 17p, 5q14, *ATM*. We removed duplications in 15q. We also removed any sCNVs in 7q34 and 14q11.2, as well as trisomy 12 events. We also removed events whose copy-number state could not be determined.

Statistical analysis

Overall burden analysis

To test the hypothesis of whether more individuals with at least one sCNV of cell fraction greater than a given cell fraction cut-off in cases vs. controls, the two-sided Fisher's Exact test was used.[3] The 95% confidence intervals were calculated using Wilson's score

interval. For the meta-analysis using each batch separately we used a one-sided Fisher's Exact test. The p values were combined using the Tippet's (minimum p value), and the Liptk's (weighted sum of p values) approaches.

Cell fraction, gene-set, length, and gene number burden analysis

To calculate the contribution of the features of gene, length, and gene number burden, we fit a mixed effect logistic regression on the case-control phenotype as the outcome variable. Let $y_i \in \{0,1\}$ be an indicator of whether the subject is diagnosed with SCZ or a control respectively. We modeled the burden as follows:

$$logit(Pr(y_i = 1)) = \beta_0 + \beta_{sex}X_{i,sex} + \beta_{LENGTH}X_{i,LENGTH}$$

$$logit(Pr(y_i = 1)) = \beta_0 + \beta_{sex}X_{i,sex} + \beta_{LENGTH}X_{i,LENGTH} + \beta_{meanCF}X_{i,meanCF}$$

$$logit(Pr(y_i = 1)) = \beta_0 + \beta_{sex}X_{i,sex} + \beta_{LENGTH}X_{i,LENGTH} + \beta_{\#genes}X_{i,\#genes}$$

where X_{LENGTH} and $X_{\#genes}$ are the sum of the length and number of genes overlapped by events of individual i, and X_{meanCF} is the mean cell fraction of the events of individual i. Inference was not altered by the sufficient statistic used to summarize cell fraction (i.e. min, max, median). In the models above we were interested on testing whether $\beta \neq 0$ for the feature of interest. The models were fit using a generalized mixed-effect model as implemented by the R package lme4[62] to account for the sample collection batches of the PGC. Statistical significance was assessed using the Satterwhite approximation to the t-test as implemented in the package lmerTest.[63]

Gene set enrichment analysis

We used a similar approach as recommended by Raychaudhri et al.[66] to control for event length and rate, which might result in false positive associations with neuronal genes. Namely, we fit the following model

$$logit(Pr(y_i = 1)) = \beta_0 + \beta_{sex}X_{i,sex} + \beta_{LENGTH}X_{i,LENGTH} + \beta_{\#sCNVs}X_{i,\#sCNVs} + \beta_{geneset}X_{geneset}$$

where the parameters are as defined the section above, but with $X_{\#sCNVs}$ is the number of sCNVs in that individual, and $X_{geneset}$ is the number of genes in an event that intersect a gene-set of interest. We then used the likelihood ratio test to test whether $\beta_{geneset} \neq 0$. We used 3 gene-sets: (1) Brain expressed genes: defined as the top 20% of brain expressed genes from the GTEx GTEx_Analysis_2017-06-05_v8_RNASeQCv1.1.9_gene_median_tpm.gct.gz (https://www.gtexportal.org/home/datasets). (2) Synaptic genes obtained from SynaptomeDB (http://metamoodics.org/SynaptomeDB/index.php). (3) High pLI genes, i.e. pLI >0.90, obtained from ExAC (file: fordist_cleaned_nonpsych_z_pli_rec_null_data.txt) (https://gnomad.broadinstitute.org/downloads).

Permutation test for enrichment of sCNV overlapping exons 1–5 of NRXN1

We used the R package regioneR[60] to randomly shuffle the 7 sCNV that overlapped *NRXN1* across the *NRXN1* locus using the randomizeRegions function, to generate a null distribution of overlaps to perform a boostrap test. We added a padding of 1Mb to the 5′ and 3′ ends of the *NRXN1* locus. After randomly shuffling the sCNV we counted how many segments overlapped exons 1–5. We repeated this procedure 10,000 times. The p value was calculated empirically by the fraction of overlaps greater than the observed 5. Since we performed 10,000 iterations our smaller possible p value was 0.0001.

Germline CNV analyses

We obtained gCNV final calls from the SCZ Phase 2 study by the PGC CNV working group.[23] We narrowed down the gCNV calls to those that were identified in the same genotype arrays that were analyzed for sCNVs. To further control for sensitivity between the methods used to call sCNVs and gCNVs we focused on gCNV events with size >100Kb. Length analysis were performed using a log-normal mixed effect model framework using sample batch as the random effect. Gene burden analysis was done with a negative binomial mixed effect model using batch as a random effect, and log(event length) as a covariate.

Breakpoint microhomology analysis

For the *NRXN1* somatic deletions, we identified the breakpoints at the single base resolution by looking for clipped reads with IGV[61] in the vicinity of discordant paired reads mapping to genomic locations that implied a larger insert size than expected. Microhomology was identified by looking at the surrounding bases of the clipped reads covering the breakpoint and looking for corresponding identical basepairs.

Characterization of the mechanism of origin was identified using the strategy described in Yang et al.[35] In brief, if there was no microhomology nor insertions >10 bp, the event was predicted to be created by non-homologous end-joining repair (NHEJ). If there was a microhomology >2 bp but <100 bp, the event was classified as alternative end joining (alt-EJ). If the microhomology was >100bp, which was not observed in this study, the event was classified as non-allelic homologous repair (NHAR).

The cell fraction of the events was estimated by identifying the breakpoints as above, and counting the number of clipped reads supporting the breakpoints from IGV images. Specifically, the number of clipped reads was divided by the sequencing depth at that site and multiplied by 2. For each event, the estimate of the cell fraction was obtained from the breakpoint with the highest coverage.

In situ Hi-C from hiPSC-derived neurons

Forebrain neurons were generated as previously described.[31] Briefly, neural precursor cells (NPCs) derived from hiPSCs with heterozygous germline deletions in the 5′-end (exons 1–2), 3′-end (exons 21–23) and from an hiPSC line with no germline deletion in NRXN1 were seeded at low density and cultured in neural differentiation medium (DMEM/F12, 1xN2, 1xB27-RA, 20 ng mL−1 BDNF (Peprotech), 20 ng mL−1 GDNF (Peprotech), 1mM dibutyryl-cyclic AMP (Sigma), 200nM ascorbic acid (Sigma) and 1 μgml−1 laminin (ThermoFisher Scientific) 1–2 days later. Cells were maintained in differentiation medium for 7.5 weeks before harvesting.

In situ Hi-C libraries were generated from 500K to 1 million cultured hiPSC-derived neurons using the Arima Hi-C kit (Arima Genomics, San Diego) per manufacturer's instructions without modifications. Briefly, *in situ* Hi-C consists of 7 steps: (1) crosslinking cells with formaldehyde, (2) digestion of the DNA using a proprietary restriction enzyme cocktail within intact nuclei, (3) filling and biotinylation of the resulting 5′-overhangs, (4) ligation of blunt ends, (5) shearing of the DNA, (6) pull down of the biotinylated ligation junctions with streptavidin beads, and (7) analyzing these fragments using paired end sequencing. The resulting Hi-C libraries were sequenced on the Illumina HiSeq1000 platform (125bp paired-end) (New York Genome Center).

Hi-C read alignment

Hi-C reads were aligned to the hg19 reference genome using bwa mem (v0.7.17-r1188) using the flags "-SP5M" ("-SP" for aligning each end of the paired end reads separately, "-5" to force always reporting the 5′ part of a chimeric read as primary).

Aligned reads were subsequently used for two different tasks: 1) variant calling with the GATK pipeline followed by HapCUT2 phasing, and 2) Hi-C matrix construction via pairtools.

Preprocessing for variant calling

Duplicate Hi-C reads were marked using Picard's MarkDuplicates (via GATK, v4.0.12.0). Bamfiles were recalibrated using the GATK BQSR (base quality score recalibration) procedure. Briefly, BaseRecalibrator was run using dbSNP build 138, the Mills +1000 Genomes gold standard indels, and the 1000 Genomes Phase I gold standard indels as reference variants. The recalibration adjustment was then applied with ApplyBQSR.

Variant calling for Hi-C analysis

Deduplicated and recalibrated Hi-C reads were then processed using the GATK (v4.0.12.0) germline short-read variant discovery pipeline. Briefly, HaplotypeCaller was run in gVCF mode (flags "-ERC GVCF") using dbSNP build 138 as a reference. Merged gVCFs then were converted to genomicsDB format with GenomicsDBImport and genotypes were called against this genomicsDB with GenotypeVCFs.

Variant quality scores were separately recalibrated for SNVs and indels via the GATK VQSR (variant quality score recalibration) procedure. Briefly, separate VQSR models were built for SNVs and indels using VariantRecalibrator, run in SNP or INDEL mode, respectively. The reference variants used for SNV quality recalibration were:

HapMap variants (v3.3): training and truth, prior of 15.

1000 Genomes "Omni" platform variants (v2.5): training and truth, prior of 12.

1000 Genomes Phase I gold standard SNPs: training only, prior of 10

dbSNP variants without 1000 Genomes (build 138, excluding sites after build 129): known, prior of 2.

The reference variants used for indel quality recalibration were:

Mills +1000 Genomes gold standard indels: training and truth, prior of 12.

The flags "–max-gaussians 2 -an QD -an MQ -an ReadPosRankSum -an FS -an SOR -an DP" were used when building the SNV recalibration model, and the flags "–max-gaussians 4 -an QD -an DP -an FS -an SOR -an ReadPosRankSum" were used when building the indel recalibration model.

The VQSR models for SNVs and indels were then applied using ApplyVQSR in SNP or INDEL mode, respectively, with a truth sensitivity filter level of 99.

Haplotype phasing for Hi-C analysis

Haplotypes were phased using HapCUT2. Briefly, recalibrated and filtered variants were separated for each sample, then HAIRS were extracted with extractHAIRS with flags "–hic 1 –indels 1". HAPCUT2 was then run with flag "–hic 1".

Each Hi-C read was then assigned to one of the two haplotype blocks called by HapCUT2 by counting how many variants that overlapped the read were part of each haplotype block. If a read overlapped multiple variants that were phased to different haplotype blocks, a majority voting system was used to assign those reads to the haplotype block that had more variants overlapping that read. If an equal number of variants from each haplotype block overlapped the read, the read was discarded from the phasing process.

Hi-C matrix construction and visualization

Hi-C matrices were constructed from mapped reads using the pairtools pipeline. Briefly, Hi-C read pairs were parsed, sorted, merged, and deduplicated. Restriction fragments were assigned to read pairs by using ''pairtools restrict'' with a restriction fragment bedfile generated using the ''digest_genome.py'' script from HiC-Pro.

Phased pairsfiles were generated by subsetting the unphased pairsfile to only those reads that were phased to a specific haplotype block.

Phased and unphased pairsfiles were used to assemble contact matrices using the ''juicer pre'' command in juicer_tools (v1.8.9), using a MAPQ threshold of 10. Phased matrices were assembled at 40 kKb resolution, while unphased matrices were assembled at 10 kKb resolution.

Unphased matrices were balanced using the KR (Knight-Ruiz) normalization implemented in juicer_tools and visualized in balanced form. Phased matrices were visualized in unbalanced form. H3K27ac ChIP-seq tracks from ENCODE (H1 neurons, Bernstein Lab, ENCODE ID ENCFF516KKW) were overlaid on the heatmaps.

Analysis of human postmortem snRNA-seq datasets

We gathered three publicly available postmortem snRNA-seq datasets from two studies.[52,53] We used publicly available annotations from both studies to identify cell types. To determine the log-normalized expression of *ABCB11* across these datasets, we normalized gene expression to the total number of transcripts sampled per cell, multiplied by 10000, added a pseudocount of 1, and log-transformed the data. We then averaged expression for each cell type for each cell type for each donor from the studies (e.g. 2 donors from the Bakken et al. and 8 neurotypical controls from Kamath et al.) in order to account for intra-individual variation. The uniform manifold approximation (UMAP) low-dimension embedding shown is taken from a previous analysis of the SN dataset.[52]

Single-molecule *in situ* hybridization (smFISH) and imaging of postmortem human nigra

Postmortem human midbrain tissues flash frozen in -80°C were cryosectioned at -15 to -20°C to make 12-micron sections on SuperFrost Plus slides. The slides were then allowed to warm up to room temperature (RT) before being placed in 4% PFA for 15 min at RT. Slides were next washed three times with 70% ethanol for 5 min followed by a 2-h 70% ethanol wash at RT. Subsequently, slides were incubated at 37°C in the Probe Hybridization buffer (Molecular Instruments) for 10 min in a humidified chamber to pre-hybridize. At this time, the probe solution was prepared by adding 0.4 pmol of each probe set (Molecular Instruments) per 100 μL of Probe Hybridization buffer and vortexed to ensure proper mixing. The Probe Hybridization buffer was then replaced by the probe solution and the slides were incubated overnight at 37°C in a humidified chamber. After 18–24 h, sections were sequentially washed for 15 min each in the following solutions at 37°C in a humidified chamber: (1) 75% Probe Wash buffer (Molecular Instruments) and 25% 5x SSCT (SSC +10% Tween 20), (2) 50% probe wash buffer and 50% 5x SSCT, (3) 25% probe wash buffer and 75% 5X SSCT, and (4) 100% 5x SSCT. The slides were then washed for 5 min at room temperature in 5x SSCT. Slides were then allowed to pre-amplify in the Probe Amplification buffer (Molecular Instruments) for 30+ minutes at RT. During this time, the hairpins (Molecular Instruments) are prepared. Approximately, 1uL of hairpin for every 100uL of final amplification solution were snap-cooled in a PCR thermocycler with the following settings: 95° for 90 s, cool to room temperature (20°C) at a rate of 3° per minute. After snap-cooling, hairpins were added to the desired volume of the amplification buffer. Slides were incubated overnight at RT in a humidified chamber. After overnight incubation, the slides are washed twice for 30 min at room temperature with 5x SSCT. An appropriate amount of Fluoromount Gold with NucBlue (Thermo Fisher) was added to the slides which then are coverslipped. Slides were stored at 4°C until imaging.

We used the following probe accession numbers: TH (NM_000360.4), CALB1 (NM_001366795), ABCB11 (NM_003742.4).

Imaging was performed with either a: DragonFly confocal scanner unit with an Andor Zyla 4.2 Plus camera (for high resolution images of DA neurons) or a Keyence BZ800XE microscope (for tiled image of overview SN). Images were acquired using either a Nikon Apo 10x objection (for the overview SN) or Nikon Apo 40x/1.15 WI objective for the (high resolution images of DA neurons).

Cell Genomics, Volume *3*

Supplemental information

Schizophrenia-associated somatic copy-number variants from 12,834 cases reveal recurrent *NRXN1* and *ABCB11* disruptions

Eduardo A. Maury, Maxwell A. Sherman, Giulio Genovese, Thomas G. Gilgenast, Tushar Kamath, S.J. Burris, Prashanth Rajarajan, Erin Flaherty, Schahram Akbarian, Andrew Chess, Steven A. McCarroll, Po-Ru Loh, Jennifer E. Phillips-Cremins, Kristen J. Brennand, Evan Z. Macosko, James T.R. Walters, Michael O'Donovan, Patrick Sullivan, Psychiatric Genomic Consortium Schizophrenia and CNV workgroup, Brain Somatic Mosaicism Network, Jonathan Sebat, Eunjung A. Lee, and Christopher A. Walsh

Supplemental Figures:

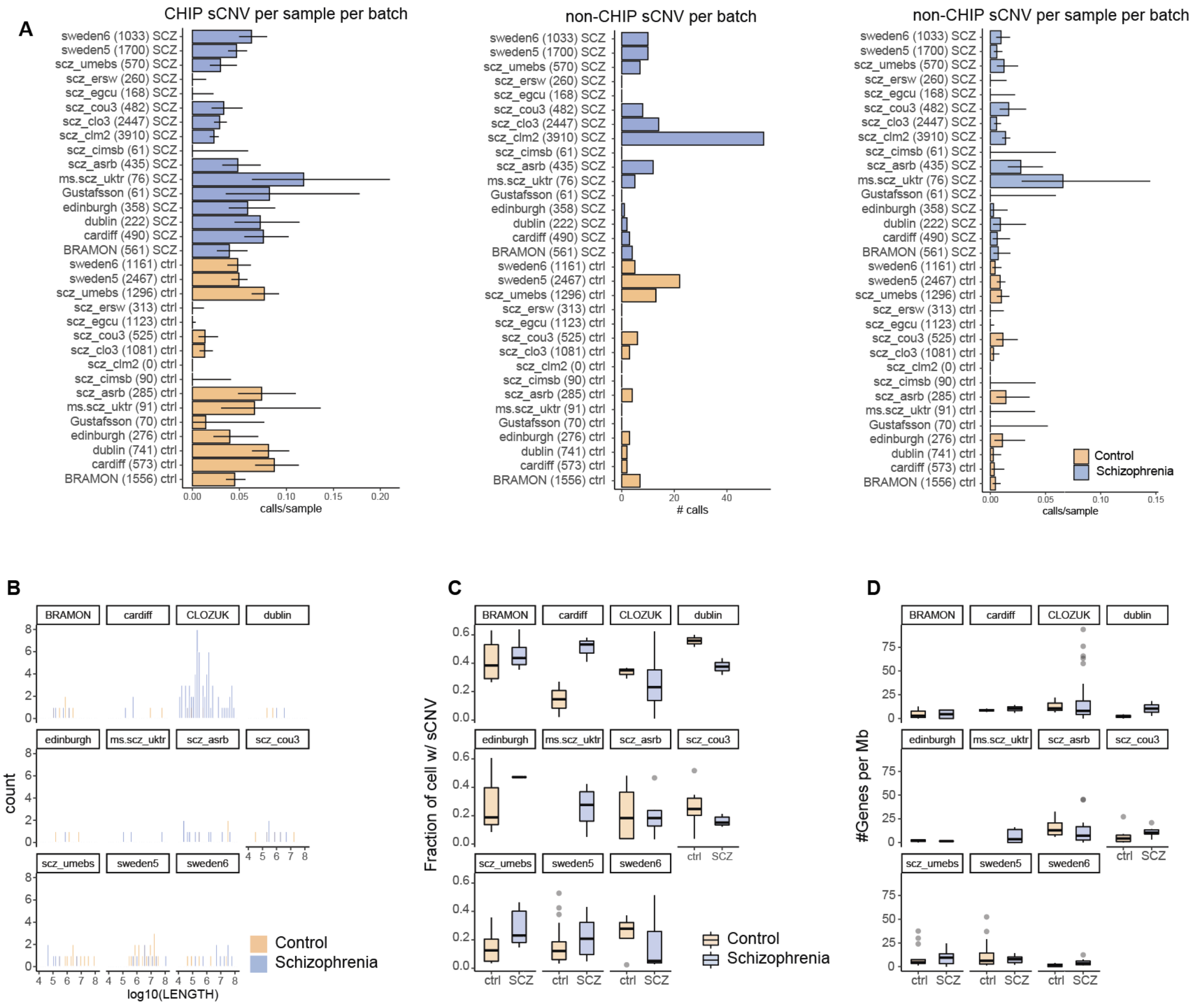

Figure S1: Characteristics of sCNVs callset across batches, related to Figure 1. A) Bar plots and forest plots of the number of sCNVs and fraction of samples with more than one sCNV in cases and controls for all batches of the data for CHIP and non-CHIP events. The number of samples on each batch is indicated in the parenthesis of the y-axis labels. The 95% confidence intervals were calculated using the Wilson's score interval with Newcombe modification. B) Histograms of sCNV length across batches for cases and controls. C) Box-plots of the fraction of cells with events (CF) in SCZ vs controls across all batches with events. D) Box-plots of the number of genes affected per megabase (Mb) in SCZ vs controls across all batches with events.

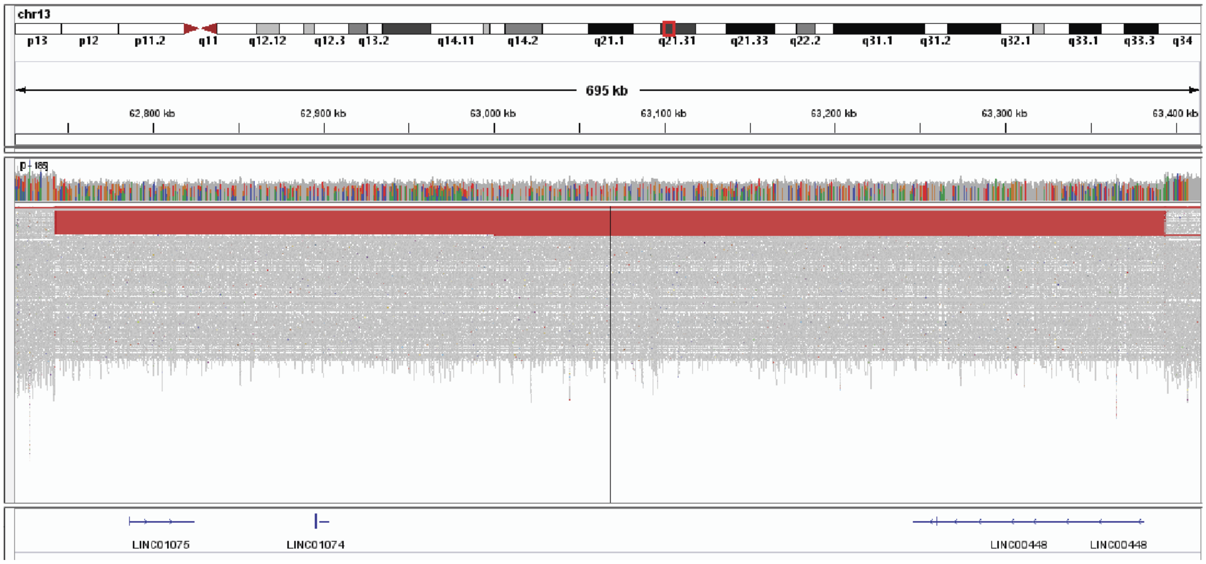

Figure S2: Further validation of sCNV, related to Figure 1. IGV plots of 9q21.11 locus. Red bars are reads with discrepant insert size corresponding to somatic deletions. The tracks from top to bottom on each panel indicates the coverage, reads mapping to that region, and RefSeq gene names respectively.

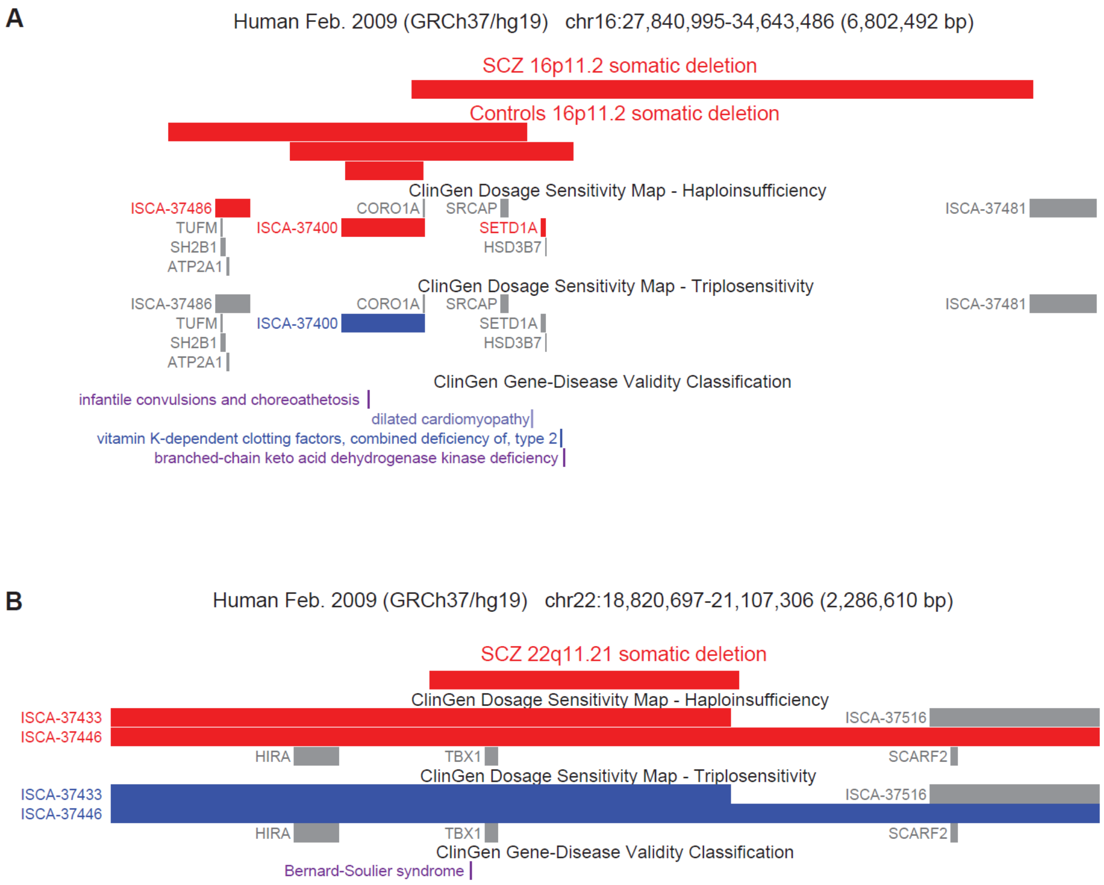

Figure S3: Somatic CNVs in 16p11.2 and 22q11.21, related to Figure 1. A) Adapted GenomeBrowser plot of 16p11.2 somatic deletions in cases and controls. Clinically relevant haploinsufficient and triplosensitive regions were annotated using the ClinGen database. Canonical 16p11.2 deletion regions are annotated by ClinGen haploinsufficiency at the proximal (ISCA-37400) and distal (ISCA-3786) sites. B) Adapted GenomeBrowser plot of 22q11.21 deletions in SCZ cases. The canonical 22q11.2 deletion regions are annotated as ISCA-37433 and ISCA-37446. For Figure A and B clinically relevant haploinsufficient and triplosensitive regions and genes were annotated using the ClinGen database. The red and blue color on in the dosage sensitivity map indicates deletions and duplications respectively. The gray color indicates that there is only moderate indication that the region/gene might be dosage sensitive. Note that *COMT* is overlapped by the 22q deletion, but is not illustrated because it is not part of the ClinGen annotation database.

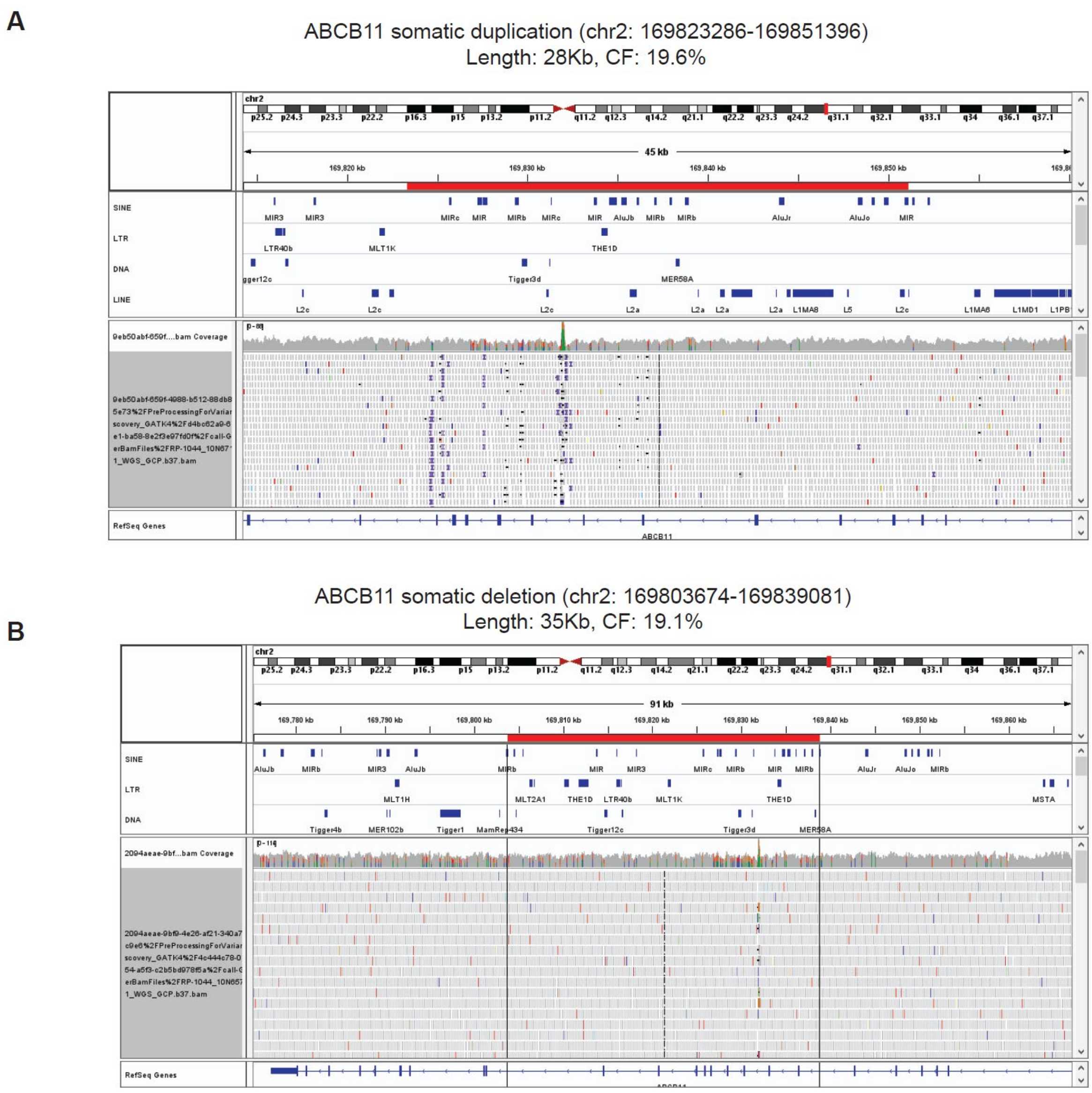

Figure S4: WGS IGV plots of *ABCB11* sCNV samples, related to Figure 4. A, B) IGV plots of *ABCB11* locus. Red bar representing the corresponding putative sCNV region. The tracks from top to bottom on each panel indicates the RepeatMasker annotation for different transposon families, coverage, and reads mapping to that region respectively.

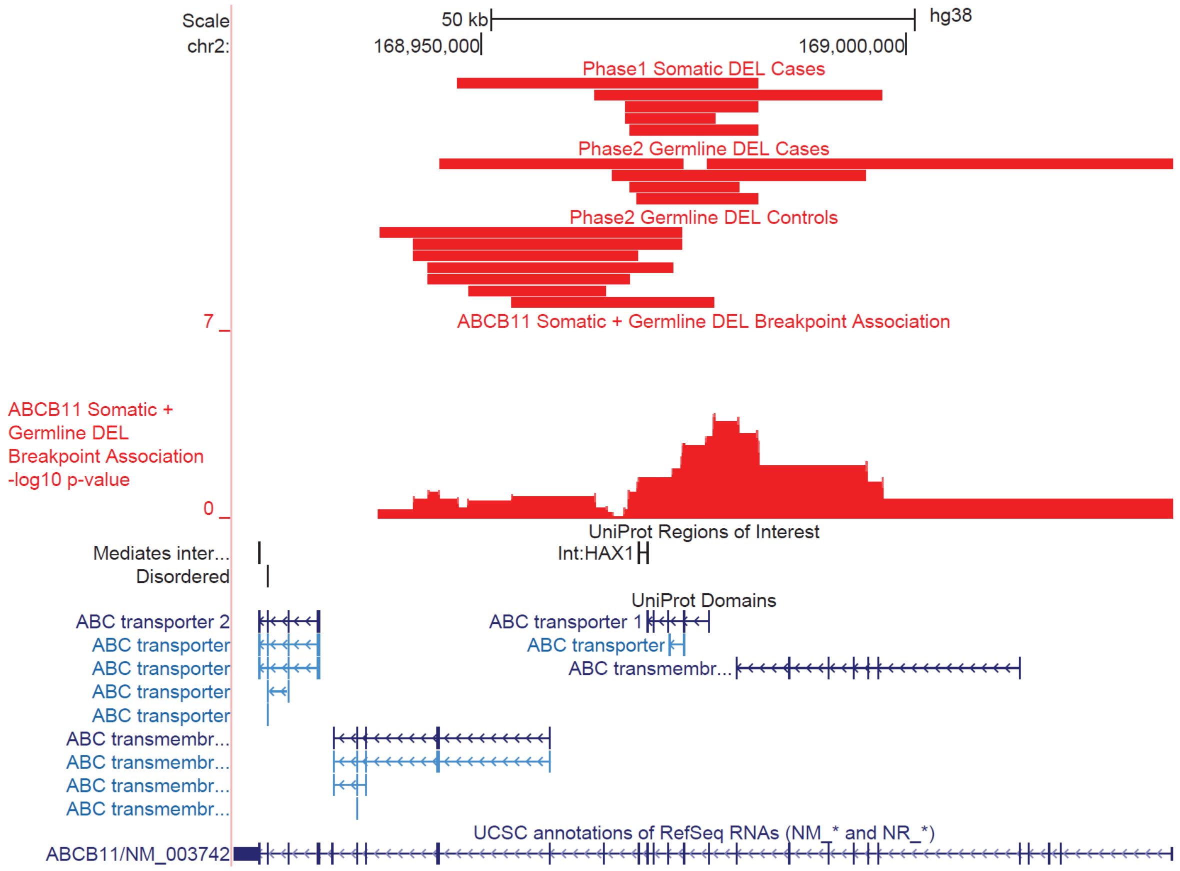

Figure S5: Somatic and Germline deletions of *ABCB11*, related to Figure 4. Adapted GenomeBrowser plot at the *ABCB11* gene locus. Association p-values were computed with logistic regression on disease status, controlling for overall CNV burden.

Table S1: sCNV burden in SCZ cases and controls by gains and losses, related to Figure 1.

Diagnosis	Total Samples	Samples w/ sCNV (#events)	% Occurrence	Samples w/ Gains (# events)	Samples w/ Loss (# events)
Schizophrenia	12,834	118 (131)	0.91	42 (48)	77 (83)
Control	11,648	60 (67)	0.52	22 (22)	39 (45)
Total	24,482	177 (198)	0.72	64 (70)	116 (127)
